# Anchoring Microbubbles on Cerebrovascular Endothelium as a New Strategy Enabling Low‐Energy Ultrasound‐Assisted Delivery of Varisized Agents Across Blood‐Brain Barrier

**DOI:** 10.1002/advs.202302134

**Published:** 2023-10-23

**Authors:** Bo Li, Yuejun Lin, Gengjia Chen, Mingyue Cai, Huihai Zhong, Zecong Xiao, Minzhao Lin, Tan Li, Yujun Cai, Xintao Shuai, Jie Ren

**Affiliations:** ^1^ Nanomedicine Research Center The Third Affiliated Hospital of Sun Yat‐sen University Guangzhou 510630 China; ^2^ Department of Medical Ultrasonic The Third Affiliated Hospital of Sun Yat‐sen University Guangzhou 510630 China; ^3^ Department of Minimally Invasive Interventional Radiology the Second Affiliated Hospital of Guangzhou Medical University Guangzhou 510260 China

**Keywords:** binding microbubbles, blood‐brain barrier, brain‐targeted delivery, cavitation effect, diagnostic ultrasound

## Abstract

The protective blood‐brain barrier (BBB) prevents most therapeutic agents from entering the brain. Currently, focused ultrasound (FUS) is mostly employed to create microbubbles that induce a cavitation effect to open the BBB. However, microbubbles pass quickly through brain microvessels, substantially limiting the cavitation effect. Here, we constructed a novel perfluoropropane‐loaded microbubble, termed ApoER‐Pep‐MB, which possessed a siloxane bonds‐crosslinked surface to increase the microbubble stability against turbulence in blood circulation and was decorated with binding peptide for apolipoprotein E receptor (ApoER‐Pep). The microbubble with tailor‐made micron size (2 µm) and negative surface charge (−30 mV) performed ApoER‐mediated binding rather than internalization into brain capillary endothelial cells. Consequently, the microbubble accumulated on the brain microvessels, based on which even a low‐energy ultrasound with less safety risk than FUS, herein diagnostic ultrasound (DUS), could create a strong cavitation effect to open the BBB. Evans Blue and immunofluorescence staining studies demonstrated that the DUS‐triggered cavitation effect not only temporarily opened the BBB for 2 h but also caused negligible damage to the brain tissue. Therefore, various agents, ranging from small molecules to nanoscale objects, can be efficiently delivered to target regions of the brain, offering tremendous opportunities for the treatment of brain diseases.

## Graphical abstract

A novel microbubble decorated with ApoER‐Pep for specific binding to the low‐density lipoprotein receptors predominantly expressed on brain microvessels is constructed. This strategy greatly boosted the diagnostic ultrasound‐elicited cavitation effect of microbubbles on the wall of brain microvessels while significantly reducing side effects for opening the blood‐brain barrier, which provided tremendous opportunities for testing varisized therapeutic/diagnostic agents against brain diseases.

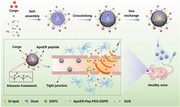

## 1 Introduction

Intracranial diseases, such as brain tumors and neurodegenerative diseases, are among the top threats to human health. Unfortunately, early diagnosis and effective therapy of brain diseases are difficult because of the presence of the blood‐brain barrier (BBB), which blocks the entry of almost 100% of large‐molecule therapeutic agents and over 98% of small‐molecule therapeutic agents into the brain.^[^ ^1^ ^]^ After highly risky surgical resection,^[^ ^2^ ^]^ the median survival of adult patients diagnosed with glioma is only 12–18 months despite great advances in medicine.^[^ ^3^ ^]^ In the realm of neurological diseases, such as Alzheimer's disease, which affects approximately one‐third of individuals over the age of 85, effective treatments remain elusive thus far.^[^ ^4^ ^]^ On the other hand, early diagnosis of brain diseases is greatly complicated because of the BBB as well. So far, contrast agents capable of crossing the BBB are not clinically available for magnetic resonance imaging (MRI) which has been widely used to detect intracranial abnormalities.

To date, two different strategies have been proposed for delivering nanoagents, encompassing both nanodrugs and medical imaging nanoprobes, into the brain.^[^ ^5^ ^]^ On one hand, nanoagents modified with targeting ligands for specific receptors on brain endothelial cells may enable receptor‐mediated transcytosis which assists them to cross the BBB.^[^ ^6^ ^]^ However, although similar methods may work well for small‐molecule agents, their effectiveness seems to be poor for nanoagents due to the nanosize impediment.^[^ ^7^ ^]^ For instance, despite decoration with transferrin, very limited amounts of drugs (less than 1%) can achieve BBB‐crossing delivery into the brain by nanocarriers.^[^ ^8^ ^]^ Therefore, the efficient delivery of nanoscale therapeutic agents, including antibodies and nanotherapeutics (e.g., Abraxane), into the brain through targeting ligand modification remains a great challenge. In contrast, irradiating microbubbles with focused ultrasound (FUS) has demonstrated effectiveness in opening the BBB.^[^ ^9^ ^]^ FUS irradiation creates mechanical forces, such as pressure and shock waves, at the endothelial surface, which may loosen the endothelial tight junction and result in the unstable expansion of microbubbles, leading to an eventual explosion, a phenomenon known as the cavitation effect.^[^ ^10^ ^]^ Although this high‐energy process may promote a burst release and diffusion of drugs at the diseased sites of interest, it is also reported to associate with serious side effects including vascular rupture to induce hemorrhage; vascular constriction to cause ischemia; protein extravasation to cause cerebral edema and/or inflammation; and mechanical forces to cause direct tissular injury and even dysfunction in visual, motor, or learning behaviors.^[^ ^11^ ^]^ In addition, the focal zone of the FUS transducer can only be designated for dimensions of up to 2–3 mm.^[^ ^5^, ^12^ ^]^ Thus, opening the BBB with FUS irradiation only results in drug penetration within a very small area, which is not suitable for treating stroke, Alzheimer's, and advanced glioma involving large areas of brain tissue. Furthermore, it may take as long as 48 h for the FUS‐opened BBB to reseal,^[^ ^13^ ^]^ raising concerns regarding the security of the central nervous system. Low‐energy ultrasound, such as diagnostic ultrasound (DUS), is a ubiquitous diagnostic imaging tool in clinics that can induce cavitation in large areas.^[^ ^14^ ^]^ When microbubbles pass through brain microvessels, DUS may create a cavitation effect, and its low acoustic energy is favorable for reducing side effects on the fragile brain.^[^ ^15^ ^]^ Unfortunately, the injected microbubbles pass through the brain microvessels so quickly that only a very small proportion participates in the DUS‐induced cavitation effect to open the BBB.^[^ ^5b^ ^]^ Moreover, microbubbles are rapidly cleared by the reticuloendothelial system (RES) after intravenous administration, leading to short half‐lives.^[^ ^16^ ^]^ Moreover, microbubbles composed of lipid bilayer membranes display high fluidity and poor colloidal stability, further shortening their half‐lives.^[^ ^17^ ^]^ For example, the three microbubbles (Optison, Definity, and SonoVue) approved for clinical applications, including liver diagnosis and vascular analysis, display half‐lives within several minutes, meaning that repeated injections are needed in order to obtain a sufficient half‐life.^[^ ^18^ ^]^ These drawbacks strongly challenge the efforts of employing DUS to achieve a strong cavitation effect for opening the BBB, and biosafety concerns exist in the event of repeated injections of microbubbles.^[^ ^14^, ^19^ ^]^ Therefore, developing a new strategy to make microbubbles stay alongside the walls of brain microvessels is of great importance because it may boost the low‐energy ultrasound‐triggered cavitation effect to open the BBB at a reduced risk of damaging fragile brain tissue.

**Scheme 1:**
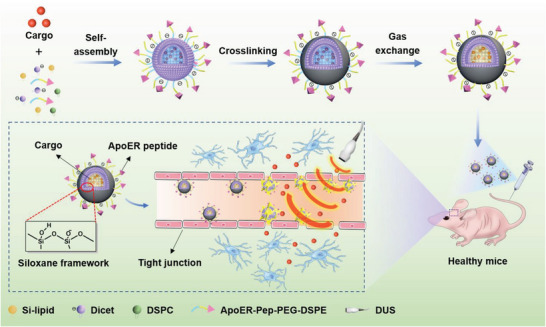
Schematic illustration of opening the BBB with ApoER‐Pep‐MB under DUS irradiation to transport varisized agents into the brain tissue.

Herein, we propose a new strategy using brain microvascular endothelium‐binding microbubbles to boost the low‐energy DUS‐induced cavitation effect for temporarily opening the BBB in a mouse model (**Scheme** **1**). A lipidic organoalkoxysilane was introduced into the microbubble because the triethoxysilyl groups can hydrolyze to generate a polyorganosiloxane, strengthening the morphological stability (Figure S1, Supporting Information). Moreover, the microbubble was decorated with a peptide (ApoER‐Pep) with the sequence (LRKLRKRLL)_2_C‐N_3_ for specific binding to the apolipoprotein E receptors predominantly expressed on brain microvessels.^[^ ^20^ ^]^ The microbubble was endowed with a micron size (≈2 µm) and high negative surface (≈−30 mV) to reduce the endocytosis by brain capillary endothelial cells (BCECs). Owing to the much lower ultrasound energy than FUS, the new strategy is expected to open the BBB without causing severe damage to the arteriovenous vessels and fragile brain tissue, which may allow for efficient and safe intracranial delivery of varisized objects, including small molecular imaging agents, antibodies, and nanoagents, for the diagnosis and treatment of brain diseases.

## 2 Results and Discussion

### 2.1 Polymer Synthesis and Microbubbles Preparation

**Figure 1:**
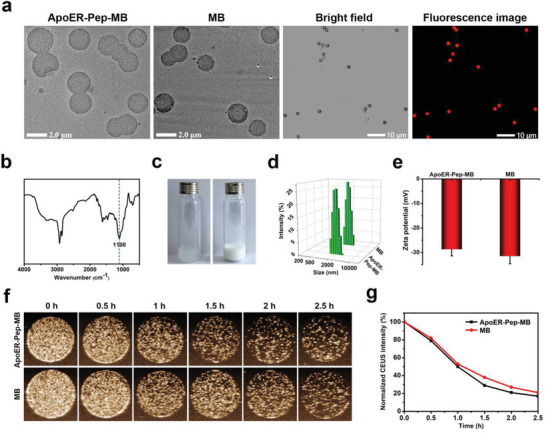
Characterization of bindable ApoER‐Pep‐MB and non‐bindable MB. a) TEM images of ApoER‐Pep‐MB and MB, bright‐field and fluorescence images of DOX‐loaded ApoER‐Pep‐MB using CLSM. b) FTIR spectrum of the ApoER‐Pep‐MB powder. Si‐O‐Si stretching band was observed at 1100 cm^−1^. c) Photographs showing gas‐free (left) and C_3_H_8_ gas‐filled (right) states of ApoER‐Pep‐MB. d) Particle sizes and e) zeta potentials for ApoER‐Pep‐MB and MB, data are expressed as mean ± SD (*n* = 3). f) CEUS images of ApoER‐Pep‐MB and MB solutions acquired at different times at room temperature. g) Quantitative analysis of ultrasonic signal intensities in f) over time using Image J software.

Brain capillary endothelial‐bindable and non‐bindable microbubbles filled with perfluoropropane (C_3_F_8_), termed ApoER‐Pep‐MB and MB, respectively, were prepared using a thin‐film hydration method based on organoalkoxysilane (Si‐lipid) and DBCO‐PEG_2k_‐DSPE synthesized in the laboratory and confirmed by ^1^H NMR analysis (Figures S1 and S2, Supporting Information). Transmission electron microscopy (TEM) and confocal laser scanning microscopy (CLSM) observations showed that ApoER‐Pep‐MB and MB both possessed spherical morphology and uniform particle size around 2 µm (**Figure** **1**a). The introduction of Si‐lipid into the microbubble provided a siloxane bond‐crosslinked surface, which may have increased the stability of the microbubble against turbulence in blood circulation. Indeed, as revealed by Fourier transform infrared spectroscopy (FTIR) analysis, the microbubble samples after the hydration process showed strong stretching bands of Si‐O‐Si around 1100 cm^−1^ (Figure 1b), and ^1^H NMR analysis showed that the methylene protons in the ethoxy group of the Si‐lipid ((CH_3_C ***H_2_*** O)_3_Si‐) almost disappeared after incubation in a pH 4 solution overnight (Figure S3, Supporting Information), indicating the hydrolysis of triethoxysilyl groups on the microbubble surface to form siloxane bonds. C_3_F_8_ was introduced into the microbubble core, and the microbubble solution changed from transparent to opaque (Figure 1c). The dynamic light scattering (DLS) measurements were consistent with the TEM observations (Figure 1d). It is worth noting that large particle size is essential for microbubbles to effectively bind to BCECs, because small particles are more prone to rapid endocytosis by endothelial cells.^[^ ^21^ ^]^ Specifically, the range of particle sizes most easily endocytosed by cells is 50–200 nm. The larger the particle size, the more difficult it is to be endocytosed by cells.^[^ ^22^ ^]^ Furthermore, due to the incorporation of the negative electrolyte lipid (Dicet), ApoER‐Pep‐MB and MB showed highly negative zeta potentials of −28.7 ± 2.7 mV and −31.5 ± 3.1 mV, respectively (Figure 1e). Since the BBB exhibits an overall negative charge owing to the expression of sialo‐glycoconju‐gates and heparan sulfate proteoglycans on the luminal surface of BCECs at systemic physiological environment, cationized particles may trigger the adsorption‐mediated endocytosis by electrostatic interactions between the positively charged moieties of the particles and negatively charged membrane surface regions of the BBB.^[^ ^23^ ^]^ Therefore, a negative surface charge can prevent BBB‐bindable microbubbles from being endocytosed by BCECs.^[^ ^24^ ^]^

Figure 1f,g and Figure S4, Supporting Information showed typical contrast‐enhanced ultrasound (CEUS) images of ApoER‐Pep‐MB and MB acquired in vitro. The two microbubbles exhibited similar signal evolution with an imaging lifetime of up to 2.5 h (Figure 1f,g), which was much longer than that of conventional liposomal microbubbles. For example, SonoVue required less than 5 min under the same conditions.^[^ ^16b^ ^]^ This could be attributed to the Si‐O‐Si crosslinking structure on the surfaces of the microbubbles, which considerably improved the morphological stability crucial for the application of microbubbles as an ultrasound contrast agent. Conventional microbubbles show poor colloidal stability in the blood stream because of the high fluidity of their lipid bilayer membranes, which hardly withstand dissociation caused by the shear stress of flowing blood.^[^ ^17^ ^]^ In view of the fact that the change in particle size could reflect the stability of the microbubbles, we further used a microfluidic chip to simulate the dynamic blood environment and investigated the stability of our tailor‐made microbubbles. The results showed that ApoER‐Pep‐MB maintained a particle size of ≈2 µm within 24 h, while microbubbles without adding Si‐lipid continued to disintegrate in the dynamic blood environment and reassembled into small nano‐sized particles or agglomerated into larger micron particles (Figure S5, Supporting Information). This result indicated that the incorporation of Si‐lipid improved the stability of the microbubbles in the bloodstream to ensure high ultrasound sensitivity even after binding to BCECs.^[^ ^25^ ^]^

### 2.2 Evaluation of Microbubble Binding to BBB In Vivo

**Figure 2:**
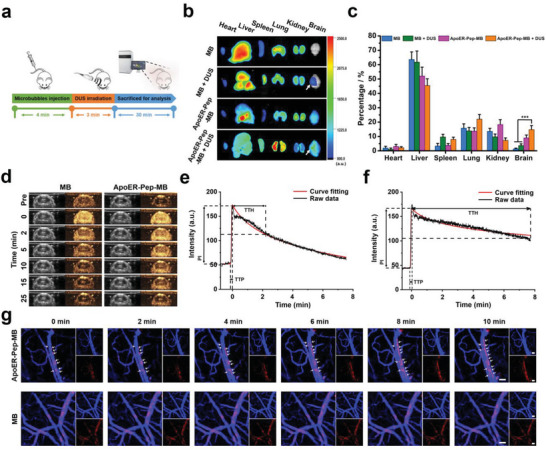
In vivo brain microvessel binding of microbubbles. a) Schematic of the study design for in vivo fluorescence imaging in mice. b) Ex vivo fluorescence imaging of major organs from mice tail vein injected with ICG‐loaded microbubbles (ApoER‐Pep‐MB or MB) with or without head‐localized DUS irradiation (3 MHz, 22.5 cycles, 3 min). White arrows indicate DUS‐irradiated areas. c) Relative fluorescence intensities of major organs from (**b**), data are expressed as mean ± SD (n = 3), ***P < 0.001 versus the MB group or the MB + DUS group. d) Brain CEUS images of mice at different time points after *i.v*. injection of MB or ApoER‐Pep‐MB. Left: gray scale; right: harmonic in each pair of images. e,f) Plotting of signal intensities from CEUS imaging against time after tail vein injection of (**e**) MB and (**f**) ApoER‐Pep‐MB, where TTP, PI, and TTH stand for time to peak, peak intensity, and time from peak to half, respectively. g) Images showing gradual binding of ApoER‐Pep‐MB or MB to microvessels of mouse brain (scale bar: 40 µm; blue: FluoSpheres carboxylate‐modified microspheres to mark the blood vessel; red: microbubbles labeled by DiI). Arrows indicate enhanced signals from ApoER‐Pep‐MB.

The biodistribution and BBB‐binding efficiency of microbubbles in mice were investigated using in vivo fluorescence imaging assay. ICG, a fluorescent agent approved by the FDA for in vivo use, with a molecular weight of 775 Da, was encapsulated in microbubbles to trace their distribution in mice (**Figure** **2**a). As shown in Figure 2b, the brain microvessel‐nonbindable MB hardly accumulated in the brain but was mostly distributed in the liver and spleen due to the preferential uptake by the RES.^[^ ^18^ ^]^ Besides, there was strong accumulation of ICG fluorescence in the lungs and kidneys of mice in all treatment groups, which might be attributed to the metabolism of free ICG. In contrast, the bindable ApoER‐Pep‐MB accumulated much more in the brain and correspondingly less in the liver and spleen. Interestingly, local DUS irradiation further enhanced brain accumulation to form a large hyperintense fluorescence area, indicating that ICG was efficiently delivered to the brain parenchyma through the opened BBB. Roughly, the brain ICG fluorescence accounted for about 14.8 ± 2.8% of the whole fluorescence intensity of the major organs (Figure 2c).

Continuous B‐mode and CEUS video clips were acquired to compare the duration of ultrasound imaging and the binding abilities of ApoER‐Pep‐MB and MB. As shown in Figure 2d, bright DUS imaging was observed in the brain after tail vein injection of microbubbles. Notably, the ultrasound imaging signal produced by ApoER‐Pep‐MB lasted much longer than that produced by MB. The changes in ultrasound signal intensity over time in the brains of mice were quantitatively analyzed (Figure 2e–f), and the perfusion parameter values of microbubbles were summarized (Table S1, Supporting Information). The raw data of the intensity‐time curves fitted well into the mathematical equation model (R^2^ > 0.94), which indicated the reliability of the perfusion parameter values calculated from the fitted equation.^[^ ^26^ ^]^ Because the ApoER‐Pep‐MB and the MB groups showed similarities in the number of microbubbles and blood flow of mice according to the undifferentiated TTP, ascending slope, and PI values (Figure 2e,f and Table S1, Supporting Information), the differences in the descending slope and TTH values implied that the reduced attenuation of the ultrasound signal in the ApoER‐Pep‐MB group was due to the binding of ApoER‐Pep to the LDL receptor.^[^ ^27^ ^]^

The BCEC‐binding capacities of the two microbubbles were compared via direct observation using intravital real‐time CLSM. Beforehand, we preliminarily verified that the incorporation of ApoER‐Pep enhanced microbubble binding to bEnd3 cells by CLSM observation (Figure S6, Supporting Information). Subsequently, the real‐time two‐photon CLSM observation of brain microvessels has provided more intuitive evidence. As shown in Figure 2g, MB passed quickly through the intracranial vessels, showing no clues to stop by. Meanwhile, the intravascular fluorescence intensity decreased by approximately 10% in 10 min, most likely due to the clearance of micron‐sized particles by the RES.^[^ ^18^ ^]^ In contrast, ApoER‐Pep‐MB effectively bound to BCECs, leading to quick accumulation of fluorescent microbubbles alongside the walls of brain microvessels after intravenous injection. Accordingly, the intravascular fluorescence remained high from 4 to 10 min after the injection of ApoER‐Pep‐MB (Figure S7, Supporting Information). Hence, 4 min postinjection was chosen as the proper time point for applying DUS to a trigger local cavitation effect to open the BBB, which ensured that the DUS also acted efficiently over a sufficient time window. The brain microvessel‐binding processes of the two microbubbles were recorded and presented in Supplementary Movies S1 and S2, Supporting Information.

### 2.3 BBB Opening and Recovery In Vivo

**Figure 3:**
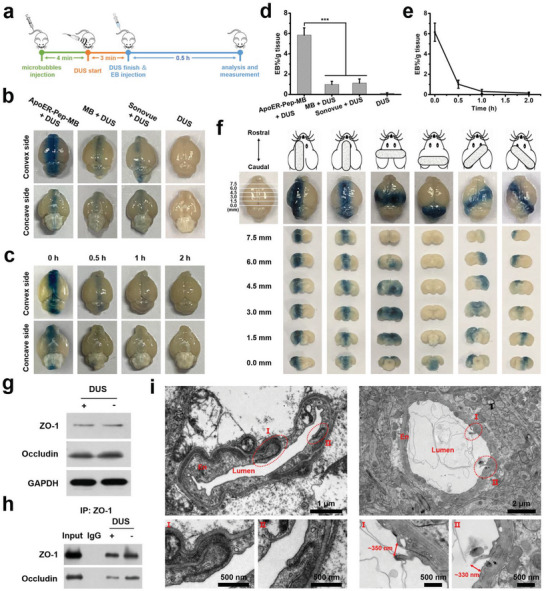
BBB opening and recovery. a) Schematic of study design for opening the BBB. b) Distribution of EB dye extravasation in the convex and concave side views of mouse brains after tail vein injection of ApoER‐Pep‐MB, MB, SonoVue,or PBS, followed by head‐localized DUS exposure. c) EB extravasation in the mouse brain at different time points after ApoER‐Pep‐MB + DUS treatment. d) Quantitative analysis of EB extravasation in mouse brains based on (**b**), data are expressed as mean ± SD (*n* = 3), ***P < 0.001 versus the MB + DUS group or the SonoVue + DUS group. e) Quantitative analysis of EB extravasation in mouse brains based on (**c**), data are expressed as mean ± SD (*n* = 3). f) EB extravasation‐indicated BBB opening directed by the DUS transducer from the surface (top) and coronal section (bottom) views of mouse brains. g) Expression levels of Occludin and ZO‐1 determined by western blotting in the presence or absence of DUS irradiation after ApoER‐Pep‐MB injection. h) Co‐immunoprecipitation (Co‐IP) of Occludin and ZO‐1 in the irradiated (DUS+) and non‐irradiated (DUS‐) brain regions of mice after injection of ApoER‐Pep‐MB. i) TEM images of brain capillaries in irradiated (right) and non‐irradiated (left) brain regions of mice after ApoER‐Pep‐MB injection. En, brain microvascular endothelial cells. Red circles indicate dense or opened tight junctions.

We assessed whether the brain microvessel‐bindable microbubble assist low‐energy DUS in opening the BBB in mice using Evans Blue dye (EB) as an indicator of BBB permeability (**Figure** **3**a). According to the concentration of SonoVue for clinical contrast‐enhanced ultrasound, 50 µL of microbubble solution (2.5 × 10^6^ mL^−1^) was injected into mice weighing around 20 g, which aligned the dosage for clinical application.^[^ ^28^ ^]^ Four minutes after the injection, DUS irradiation was performed for 3 min. The extent of EB staining on the convex and concave sides of the brain was shown in Figure 3b. No appreciable EB staining was observed in the brains of mice in the control group. In contrast, mice receiving ApoER‐Pep‐MB, MB, and SonoVue and then undergoing DUS irradiation of the head showed clear EB staining in their brain parenchyma. Among the three groups, mice subjected to DUS irradiation of the head after injecting ApoER‐Pep‐MB showed the most intense EB staining. Consistently, quantitative UV‐vis analysis of EB extracted from whole brain tissue indicated that EB extravasation in the ApoER‐Pep‐MB treatment group was more than five times higher than that in the MB and the SonoVue treatment groups (Figure 3d and Figure S8, Supporting Information). Obviously, anchoring the microbubble to the cerebrovascular endothelium enabled DUS to open the BBB much more effectively because a much stronger cavitation effect on the walls of brain microvessels was achievable in this case. The duration of BBB opening caused by DUS‐mediated cavitation of ApoER‐Pep‐MB was further evaluated. As shown in Figure 3c, the BBB permeability gradually decreased over time after DUS irradiation. For example, brain EB staining became much weaker at 0.5 h and was hardly detectable at 1 h. Quantification of EB extravasation obtained consistent results. The opening of the BBB by the DUS‐triggered cavitation effect of ApoER‐Pep‐MB completely restored its initial tightness within 2 h (Figure 3e).

The above results indicate that the low‐density lipoprotein receptors predominantly expressed on the brain microvessels provided an anchoring site for the ApoER‐Pep‐decorated microbubbles,^[^ ^20^ ^]^ which drove microbubble accumulation on the walls of brain microvessels to boost the local cavitation effect. Consequently, the BBB of mice can be opened using safe low‐energy DUS. Compared with studies in which the recovery time of the opened BBB was 24 h, and 48 h, or even 5 d, the quick recovery of the thus‐opened BBB in our study would reduce the risks of exposing the central nervous system for a long time.^[^ ^13^, ^29^ ^]^

Furthermore, we studied the relationship between the placement direction of the transducer and EB staining from the surface or coronal section views of the brain. As shown in Figure 3f, EB staining in the brain tissues of mice completely correlated with the locations receiving ultrasound irradiation. These results indicate that our new strategy may selectively open the BBB wherever treatment is needed, providing a readily attainable means for physically guided targeting of diseased brain tissues.

Next, tight junction protein expression was measured to further demonstrate the BBB opening by DUS‐assisted ApoER‐Pep‐MB. The expression levels of Occludin and ZO‐1 protein complexes in tight junction components of the irradiated and non‐irradiated brain region were analyzed by western blotting. As shown in Figure 3g, there were no significant differences in the expression of Occludin and ZO‐1 between the irradiated and non‐irradiated regions. However, the results of the co‐immunoprecipitation (Co‐IP) assay showed that the interaction between Occludin and ZO‐1 decreased in the brain region treated with DUS, indicating opening of the BBB (Figure 3h).^[^ ^30^ ^]^ Besides, the size of the paracellular gaps generated by the DUS‐induced BBB opening was also investigated using TEM. As shown in Figure 3i, there was a dense tight junction between the vascular endothelial cells in the non‐irradiated region. In contrast, a 300–400 nm gap appeared between the vascular endothelial cells in the irradiated brain region.

### 2.4 Biosafety of Opening the BBB

**Figure 4:**
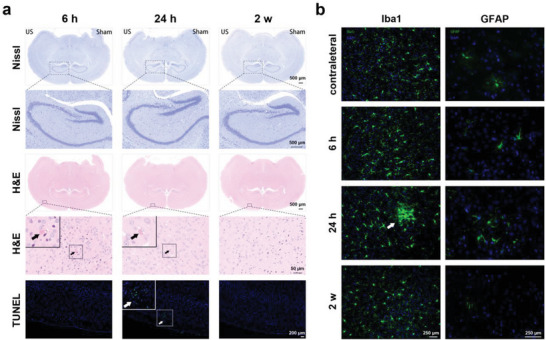
Biosafety assessment in vivo. a) Images showing Nissl, H&E, and TUNEL staining of mouse brain tissues harvested 6 h, 24 h, and 2 w after ApoER‐Pep‐MB + DUS treatment. Black arrows indicate slight extravasation of erythrocytes, and white arrows indicate apoptotic cells in small areas. b) Expressions of Iba1 in microglia and GFAP in astrocytes in mouse brain tissues harvested 6 h, 24 h, and 2 w after ApoER‐Pep‐MB + DUS treatment. White arrows indicate mild activation of microglial cells.

The biosafety of DUS‐assisted ApoER‐Pep‐MB application in vivo was assessed based on histological changes in the brain and other major organs of the mice. As shown in **Figure** **4**a, Nissl staining of the DUS‐irradiated brain showed that no “dark” neuron in the cortex and hippocampus was caused by the ApoER‐Pep‐MB + DUS treatment, indicating negligible neuron degeneration.^[^ ^31^ ^]^ Moreover, hematoxylin and eosin (H&E) staining showed only slight extravasation of erythrocytes at 6 h and 24 h after DUS irradiation, and the TUNEL assay only found very few apoptotic cells in the DUS‐irradiated area at 24 h. Then, the astrocytic marker GFAP (glial fibrillary acidic protein) and microglial cytoplasmic marker Iba1 (ionized calcium‐binding adaptor molecule 1) were detected to show the inflammation caused by the mechanical forces of ultrasonic cavitation.^[^ ^32^ ^]^ As shown in Figure 4b, little astrogliosis was detected using the GFAP‐specific antibody, and mild activation of microglial cells was observed from the Iba1 reactivity 24 h postsonication. Moreover, complete recovery to the initial status was observed within 2 weeks. These changes in microglial and astrocyte activation were in line with the H&E and TUNEL results, suggesting a temporary sterile inflammation caused by the extravasated erythrocytes. H&E staining of major organs from mice receiving various treatments showed no significant pathological changes (Figure S9, Supporting Information). In clinical practice, the prognosis of a small intracerebral hemorrhage is generally excellent, and these patients usually do not require surgery.^[^ ^33^ ^]^ Therefore, the DUS‐assisted ApoER‐Pep‐MB application in vivo did not result in irreversible inflammation or tissue damage.

### 2.5 BBB‐Crossing Delivery of Varisized Therapeutic Agents

In view of the fact that the above results confirmed high BBB‐opening efficiency and high biosafety of DUS‐assisted ApoER‐Pep‐MB, we then evaluated the effectiveness of using this new strategy to deliver varisized therapeutic agents into the brain tissue. It is worth noting that the agents were encapsulated into the interior of the microbubble, and then the triethoxysilyl groups were hydrolyzed to form a siloxane bond‐crosslinked surface. Thus, the agents wrapped in the interior have little effect on the surface potential of the microbubbles. On the other hand, although the size of ApoER‐Pep‐MB may increase after loading with varisized agents, it usually does not exceed 10%.^[^ ^34^ ^]^ Therefore, the change in size or zeta potential of the agent‐loaded ApoER‐Pep‐MB did not affect its accumulation in vivo.

**Figure 5:**
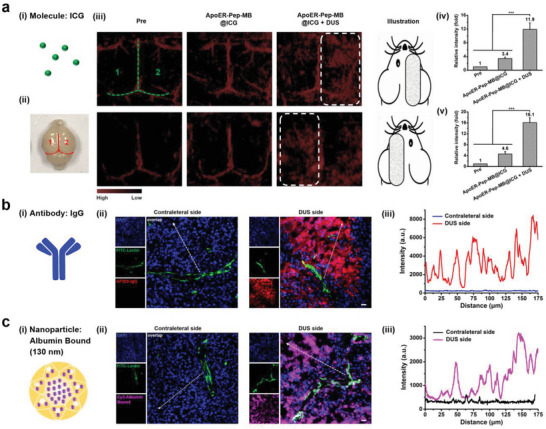
Delivery of varisized therapeutic agents into the brain via DUS‐triggered cavitation effect of ApoER‐Pep‐MB. a) Delivery of ICG (i) into the mouse brain monitored by in vivo PA imaging (iii), numbers in green color mark the left and right hemispheres while dashed lines in green color mark the three blood vessels shown as red lines in (ii). White dashed lines indicate areas with DUS irradiation. (iv‐v) Quantitative analysis of normalized PA signal intensities in the right (iv) and left (v) hemispheres compared to mice without ApoER‐Pep‐MB@ICG (Pre) injection, data are expressed as mean ± SD (*n* = 3), ***P < 0.001. b) Delivery of IgG antibody (i) into the brain revealed by CLSM imaging (ii) of brain tissue sections in the contralateral (left) or DUS (right) side (scale bar: 25 µm; blue: nuclei stained with DAPI; green: blood vessels stained using FITC‐lectin; red: IgG labeled by AF555), and quantitative analysis (iii) of AF555 fluorescence intensities in points from blood vessel to deep parenchyma indicated by white dashed arrows (ii). c) Delivery of 130 nm Abraxane (i) into brain revealed by CLSM imaging (ii) of brain tissue sections in the contralateral (left) or DUS‐irradiated (right) side (scale bar: 25 µm; blue: nuclei stained with DAPI; green: blood vessels stained with FITC‐lectin; pink: Albumin Bound labeled by Cy3), and quantitative analysis (iii) of Cy3 fluorescence intensities in points from blood vessel to deep parenchyma indicated by white dashed arrows (ii).

First, ICG was selected as a model drug to indicate changes in BBB permeability using in vivo photoacoustic (PA) imaging (**Figure** **5**a(i)).^[^ ^35^ ^]^ As shown in Figure 5a(iii), the PA signal in the blood vessels of the brain (outlined in Figure 5a(ii)) was enhanced after injecting ICG‐loaded bindable microbubble (ApoER‐Pep‐MB@ICG) into mice via the tail vein. The hemisphere displayed much higher PA intensity at 0.5 h after exposure to DUS than the contralateral hemisphere without irradiation. The average PA intensity of the right (Figure 5a(iv)) or left (Figure 5a(v)) hemisphere increased by more than three times upon DUS irradiation, implying successful BBB‐crossing delivery of ICG into the brain tissue.

In consideration that antibodies such as rituximab (Rituxan^®^) and bevacizumab (Avastin^®^) are bringing about new opportunities for treating central nervous diseases,^[^ ^36^ ^]^ we explored whether antibodies with much bigger sizes could be transported into brain tissue with our BBB‐opening strategy. Mouse IgG with a molecular weight of 150 kDa (Figure 5b(i)) was chosen as an analogue to therapeutic monoclonal antibodies. After injecting microbubbles encapsulating AF555‐labeled IgG (ApoER‐Pep‐MB@IgG) into mice via the tail vein, we applied DUS irradiation to one cerebral hemisphere while leaving the contralateral side non‐irradiated. According to the CLSM images of the brain tissue sections pretreated with phosphate‐buffered solution (PBS) perfusion, IgG did not cross the lectin‐FITC‐labeled brain microvessels in the hemisphere without head DUS irradiation. However, the DUS‐irradiated region of another hemisphere exhibited bright red fluorescence, indicating that IgG was efficiently delivered across the BBB and diffused into the brain parenchyma (Figure 5b(ii)). The fluorescence signal intensities of the IgG gradients from the blood vessels to the parenchyma in the DUS‐applied region were markedly higher than those in the non‐DUS‐irradiated region (Figure 5b(iii)), indicating the penetration of IgG with the aid of the low‐energy DUS‐mediated cavitation effect of ApoER‐Pep‐MB@IgG.

Finally, we explored the efficiency of delivering larger nanodrugs into the brain parenchyma using our BBB‐opening strategy. Abraxane, a clinically available albumin‐bound formulation of paclitaxel with a diameter of 130 nm (Figure 5c(i) and Figure S10, Supporting Information), was labeled with Cy3 and encapsulated into the microbubble. After tail vein injection of abraxane‐encapsulated microbubbles, DUS irradiation was applied to one hemisphere. As shown in Figure 5c(ii), the brain tissue exposed to DUS exhibited strong pink fluorescence of Abraxane, whereas the non‐DUS‐irradiated tissue of the contralateral hemisphere showed negligible pink fluorescence. The fluorescence intensities indicating the Abraxane gradient distribution from the blood vessels to the deep parenchyma were much higher in the DUS‐irradiated tissue (Figure 5c(iii)), which strongly demonstrated that Cy3‐labeled Abraxane could efficiently enter the brain parenchyma through the thus‐opened BBB.

### 2.6 BBB‐Crossing Delivery of Varisized MRI Contrast Agents

**Figure 6:**
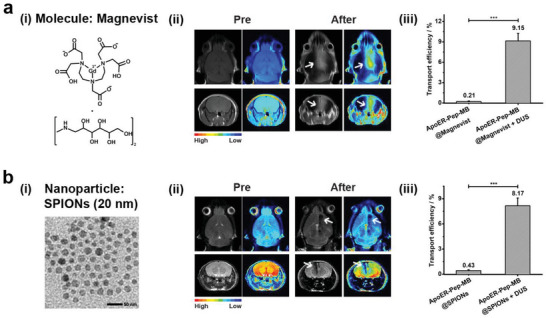
Delivery of varisized MRI contrast agents into the brain via DUS‐triggered cavitation effect of ApoER‐Pep‐MB. a) Delivery of Magnevist (i) into the mouse brain monitored by T1‐weighted MR imaging (ii) from the transverse (top panel) and the coronal (bottom panel) sectional views with white arrows indicating T1 signal‐enhanced areas. (iii) The percentage of Gd accumulated in the brain after tail‐vein injection of ApoER‐Pep‐MB@Magnevist into mice with or without head‐localized DUS irradiation, as measured by ICP‐AES (*n* = 3, ***P < 0.001). b) Delivery of water‐soluble 20 nm SPIONs (TEM image in (i)) into the mouse brain monitored by T2‐weighted MR imaging (ii) from the transverse (top panel) and the coronal (bottom panel) sectional views with white arrows indicating T2 signal‐decreased area, and (iii) percentage of Fe accumulated in the brain after tail‐vein injection of ApoER‐Pep‐MB@SPIONs into mice with or without head‐localized DUS irradiation measured by AAS (*n* = 3, ***P < 0.001).

MRI is one of the most powerful imaging techniques for clinical diagnosis of various diseases. However, the lack of BBB‐crossing MRI contrast agents greatly limits their application in the diagnosis of intracranial diseases. Even the most widely used MRI contrast agent, Magnevist, which has a molecular weight of 938 Da (**Figure** **6**a(i)), cannot cross the BBB.^[^ ^7a^ ^]^ Therefore, we encapsulated this clinically available T1‐weighted contrast agent into ApoER‐Pep‐MB and performed in vivo MRI scans to evaluate whether Magnevist could be delivered into the brain tissue using our new strategy. As shown in Figure 6a(ii), the sectional views of both coronal and transverse planes acquired prior to or at 0.5 h after the DUS irradiation showed much increased T1‐weighted MRI intensities in the irradiated area of mice receiving ApoER‐Pep‐MB@Magnevist. Notably, the transverse and coronal planes of the mouse irradiated region showed 146% and 133% increases, respectively, in T1‐weighted signal intensity (Figure S11a–d, Supporting Information). In addition, the Gd ions extracted from the whole brain tissue using a mixture of HNO_3_ and HCl (v/v = 3:1) were subjected to the measurement of ICP‐AES, which showed that 9.15 ± 1.1% of the injected Magnevist was delivered into the brain (Figure 6a(iii)). Because a BBB‐crossing MRI contrast agent is not yet available in the clinic, our strategy could be helpful for developing the next generation of BBB‐crossing MRI contrast agents to enhance precise diagnosis of brain disease in the future.

In view of superparamagnetic iron oxide nanocrystals (SPIONs) as intensively investigated MRI T2 contrast agents,^[^ ^37^ ^]^ we synthesized water‐soluble SPIONs with a diameter of 20 nm and then tried to deliver them into the brain using ApoER‐Pep‐MB (Figure 6b(i) and Figure S12a, Supporting Information). After ApoER‐Pep‐MB@SPIONs were injected via the tail vein, head‐localized DUS irradiation was applied and in vivo T2‐weighted MRI scan was performed. As shown in Figure 6b(ii), before localized DUS irradiation, the mouse brain presented a hyperintense area on the T2WI images after the administration of ApoER‐Pep‐MB@SPIONs. In contrast, at 0.5 h after DUS exposure, the irradiated area exhibited an obvious decrease in T2‐weighted signal intensity. Quantitative analysis showed that the normalized signal intensity of the irradiated area decreased to 72% and 83% in the sectional views of the transverse and coronal planes, respectively (Figure S12b–e, Supporting Information). In line with the MRI data, the Prussian blue staining assay of brain tissue sections from mice revealed that SPIONs effectively crossed the BBB and diffused deeply (Figure S12f, Supporting Information). In addition, we extracted the Fe ions from the whole brain tissue and measured them with AAS, which showed that 8.17 ± 0.92% of the administered SPIONs were delivered into the brain (Figure 6b(iii)).

## 3 Conclusions

The blood‐brain barrier, which protects the central nervous system, is also the greatest hurdle for the early diagnosis and effective therapy of brain diseases. To address this challenge, we proposed a novel approach of anchoring the microbubbles onto the brain microvessels. We aimed to utilize safe, low‐energy DUS to induce cavitation effects in the microbubbles, leading to substantial opening in the BBB of mice. In other words, the microbubbles were decorated with ApoER‐Pep to specifically bind to low‐density lipoprotein receptors predominantly expressed on BCECs, instead of arteries or veins. Thus, this novel strategy greatly boosts the low‐energy DUS‐elicited cavitation effect of microbubbles on the walls of brain microvessels while remarkably reducing side effects. The results showed that this method for opening the BBB was highly effective, by which varisized agents, including molecular imaging agents, antibodies, and even nanoparticles could be ferried into the brain tissue at transport efficiencies of over 8%. More importantly, owing to the safety features, including the low ultrasound irradiation energy and precise cavitation effects on the walls of brain microvessels, this strategy is favorable for reducing damage to fragile brain tissues. Overall, this new type of BBB‐bindable microbubble provides tremendous opportunities for testing varisized therapeutic/diagnostic agents against brain diseases in vivo.

## 4 Experimental Section

### Animal Ethics Statement

All surgical interventions in animal care were approved by the Institutional Animal Care and Use Committee of the Sun Yat‐sen University, Guangzhou, China, and performed in accordance with the experimental animal care guidelines. The assigned approval number of the ethical application of animal experiments is C2021‐0972XS.

### Statistical Analysis

Statistical analysis was carried out by SPSS software, version 22.0 (SPSS Inc.). All statistical tests were two‐tailed. The results from at least three independent samples were expressed as mean ± SD (standard deviation). Results from multiple groups were compared using Analysis of Variance (ANOVA). The least significant difference (LSD) test was performed for pairwise comparisons following ANOVA. *P* < 0.05 was considered statistically significant.

## Conflict of Interest

The authors declare no conflict of interest.

## Supporting information

Supplemental Information

Supplemental Movie 1

Supplemental Movie 2

## Data Availability

The data that support the findings of this study are available from the corresponding author upon reasonable request.
