# Supplementary material for: Anchoring Microbubbles on Cerebrovascular Endothelium as a New Strategy Enabling Low‐Energy Ultrasound‐Assisted Delivery of Varisized Agents Across Blood‐Brain Barrier
Source: Adv Sci (Weinh). 2023 Oct 23;10(33):2302134. doi: 10.1002/advs.202302134 (PMC10667842; doi:10.1002/advs.202302134)
Supplement: Supplementary file 1 — Supplemental Information [file ADVS-10-2302134-s002.pdf]

ADVANCED SCIENCE
Open Access

Supporting Information

for *Adv. Sci.*, DOI 10.1002/advs.202302134

Anchoring Microbubbles on Cerebrovascular Endothelium as a New Strategy Enabling Low-Energy Ultrasound-Assisted Delivery of Varisized Agents Across Blood-Brain Barrier

Bo Li, Yuejun Lin, Gengjia Chen, Mingyue Cai, Huihai Zhong, Zecong Xiao, Minzhao Lin, Tan Li, Yujun Cai, Xintao Shuai* and Jie Ren**

Supplementary Information for

Anchoring microbubbles on cerebrovascular endothelium as a new strategy enabling low-energy ultrasound-assisted delivery of varisized agents across blood-brain barrier

Bo Li[a,#], Yuejun Lin[b,#], Gengjia Chen[a], Mingyue Cai[c], Huihai Zhong[a], Zecong Xiao[a,*], Minzhao Lin[a], Tan Li[a], Yujun Cai[a], Xintao Shuai[a,*] and Jie Ren[b,*]

[a] Nanomedicine Research Center, The Third Affiliated Hospital of Sun Yat-sen University, Guangzhou 510630, China

[b] Department of Medical Ultrasonic, The Third Affiliated Hospital of Sun Yat-sen University, Guangzhou 510630, China

[c] Department of Minimally Invasive Interventional Radiology, the Second Affiliated Hospital of Guangzhou Medical University, Guangzhou 510260, China

[#]These authors contributed equally.

*Corresponding authors:

Xintao Shuai, E-mail: shuaixt@mail.sysu.edu.cn (ORCID: 0000-0003-4271-0310);

Jie Ren, E-mail: renjieguangzhou@126.com;

Zecong Xiao, E-mail: xiao92826@hotmail.com.

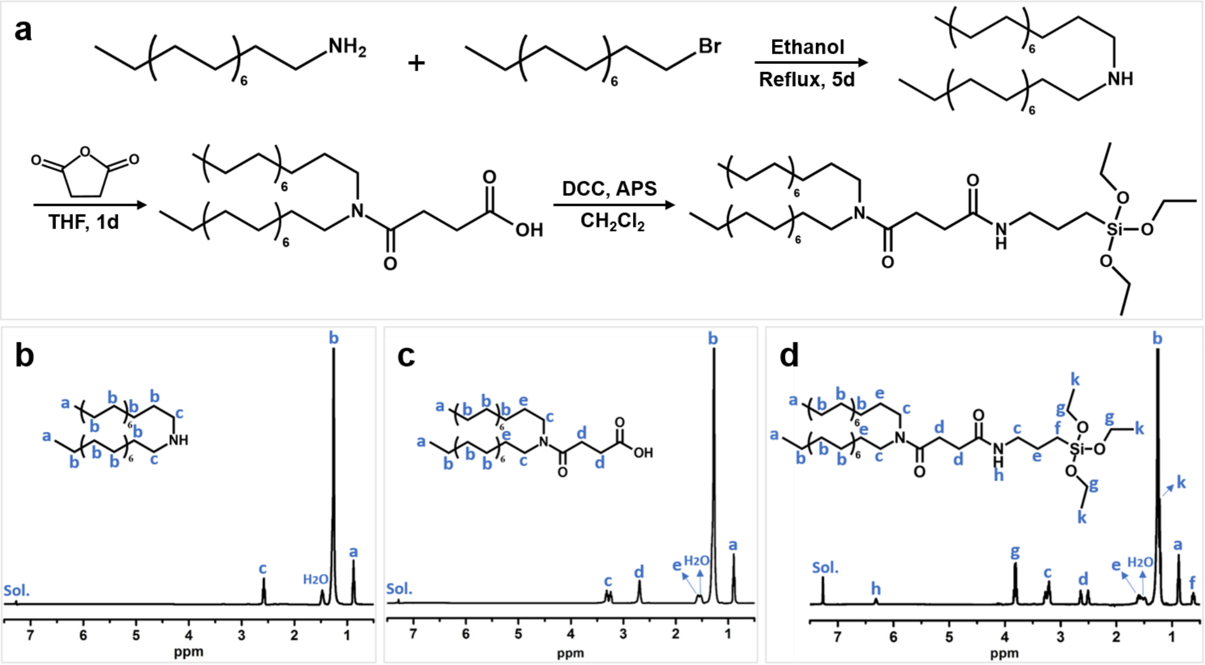

Figure S1. Synthesis of Si-lipid. (**a**) Synthetic approaches for Si-lipid and ^{1}H NMR spectra of (**b**) dihexadecylamine, (**c**) N, N'-dihexadecylsuccinamic acid, and (**d**) Si-lipid in $CDCl_3$.

^{1}H NMR spectrum of dihexadecylamine in $CDCl_3$ was shown in **Figure S1b**. The characteristic peaks of dihexadecylamine matched well with the expected chemical shifts as follow: ^{1}H NMR (400 MHz, $CDCl_3$, 298 K.): δ (ppm) = 0.89 ppm ($C\boldsymbol{H_3}(CH_2CH_2)_6CH_2CH_2CH_2NH$-, a), 1.31 ppm ($CH_3(C\boldsymbol{H_2}C\boldsymbol{H_2})_6C\boldsymbol{H_2}C\boldsymbol{H_2}CH_2NH$-, b), 2.59 ppm ($CH_3(CH_2CH_2)_6CH_2CH_2C\boldsymbol{H_2}NH$-, c).

^{1}H NMR spectrum of N, N'-dihexadecylsuccinamic acid in $CDCl_3$ was shown in **Figure S1c**. The characteristic peaks of N, N'-dihexadecylsuccinamic acid matched well with the expected chemical shifts as follow: ^{1}H NMR (400 MHz, $CDCl_3$, 298 K.): δ (ppm) = 0.89 ppm ($C\boldsymbol{H_3}(CH_2CH_2)_6CH_2CH_2CH_2$-, a), 1.27 ppm ($CH_3(C\boldsymbol{H_2}C\boldsymbol{H_2})_6C\boldsymbol{H_2}CH_2CH_2$-, b), 1.58 ppm ($CH_3(CH_2CH_2)_6CH_2\boldsymbol{CH_2}CH_2$-, e), 2.68 ppm (-$C\boldsymbol{H_2}C\boldsymbol{H_2}COOH$, d), 3.16-3.38 ppm ($CH_3(CH_2CH_2)_6CH_2CH_2C\boldsymbol{H_2}$-, c).

^{1}H NMR spectrum of Si-lipid in $CDCl_3$ was shown in **Figure S1d**. The characteristic peaks of Si-lipid matched well with the expected chemical shifts as follow: ^{1}H NMR (400 MHz, $CDCl_3$, 298 K.): δ (ppm) = 0.62 ppm (-CONH-$CH_2CH_2C\boldsymbol{H_2}$-Si-$(OCH_2CH_3)_3$, f), 0.88 ppm ($C\boldsymbol{H_3}(CH_2CH_2)_6CH_2CH_2CH_2$-, a), 1.22 ppm (-CONH-$CH_2CH_2CH_2$-Si-$(OCH_2C\boldsymbol{H_3})_3$, k), 1.26 ppm ($CH_3(C\boldsymbol{H_2}C\boldsymbol{H_2})_6C\boldsymbol{H_2}CH_2CH_2$-, b), 1.60 ppm ($CH_3(CH_2CH_2)_6CH_2\boldsymbol{CH_2}CH_2$-, e), 2.45-2.67 ppm (-$C\boldsymbol{H_2}C\boldsymbol{H_2}CONH$-, d), 3.16-3.30 ppm ($CH_3(CH_2CH_2)_6CH_2CH_2C\boldsymbol{H_2}$-, c), 3.80 ppm (-CONH-$CH_2CH_2CH_2$-Si-$(OC\boldsymbol{H_2}CH_3)_3$, g), 6.31 ppm (-CON$\boldsymbol{H}$-$CH_2CH_2CH_2$-, h).

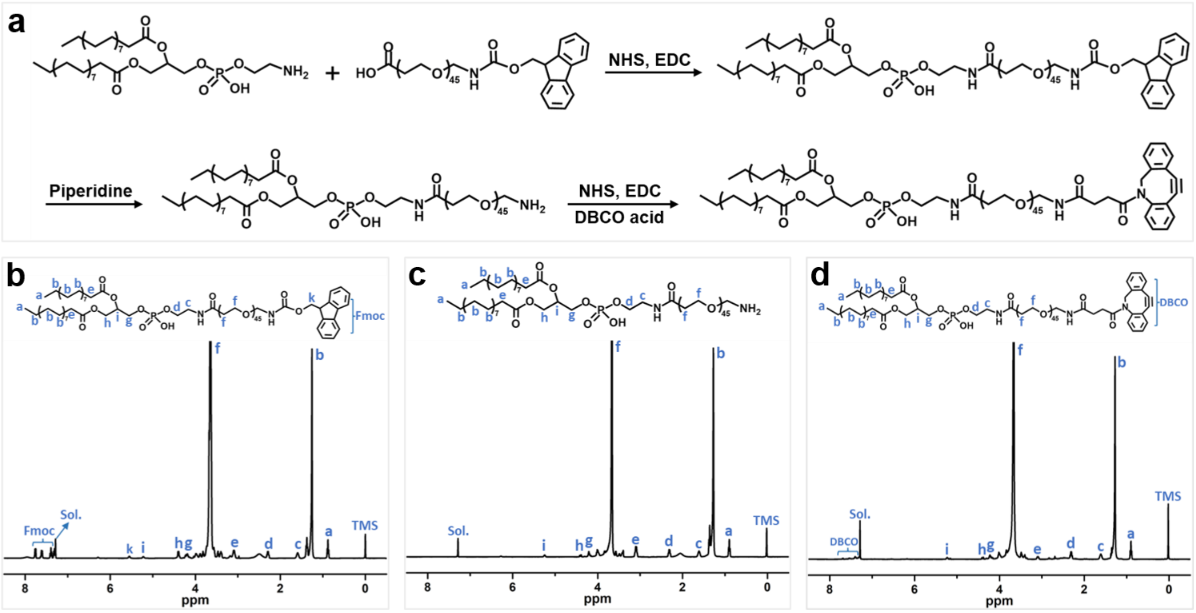

Figure S2. Synthesis of DBCO-PEG$_{2k}$-DSPE. (**a**) Synthetic approaches for DBCO-PEG$_{2k}$-DSPE and ^{1}H NMR spectra of (**b**) Fmoc-PEG$_{2k}$-DSPE, (**c**) NH_2-PEG$_{2k}$-DSPE, and (**d**) DBCO-PEG$_{2k}$-DSPE in $CDCl_3$.

^{1}H NMR spectrum of Fmoc-PEG$_{2k}$-DSPE in $CDCl_3$ was shown in **Figure S2b**. The characteristic peaks of Fmoc-PEG$_{2k}$-DSPE matched well with the expected chemical shifts as follow: ^{1}H NMR (400 MHz, $CDCl_3$, 298 K.): δ (ppm) = 0.87 ppm ($C\boldsymbol{H_3}CH_2(CH_2CH_2)_7CH_2COO$- of DSPE, a), 1.26 ppm ($CH_3C\boldsymbol{H_2}(C\boldsymbol{H_2}C\boldsymbol{H_2})_7CH_2COO$- of DSPE, b), 1.59 ppm (-$OCH_2C\boldsymbol{H_2}NHCO$- of DSPE, c), 2.28 ppm (-$OC\boldsymbol{H_2}CH_2NHCO$- of DSPE, d), 3.08 ppm ($CH_3CH_2(CH_2CH_2)_7C\boldsymbol{H_2}COO$- of DSPE, e), 3.64 ppm (-NHCO-$(C\boldsymbol{H_2}C\boldsymbol{H_2}O)_{45}$- of PEG, f), 4.21 ppm (-$COOCH_2CH(OCOCH_2$-$)C\boldsymbol{H_2}O$- of DSPE, g), 4.38 ppm (-$COOC\boldsymbol{H_2}CH(OCOCH_2$-$)CH_2O$- of DSPE, h), 5.21 ppm (-$COOCH_2C\boldsymbol{H}(OCOCH_2$-$)CH_2O$- of DSPE, i), 5.55 ppm (-$NHCOOC\boldsymbol{H_2}$- of Fmoc, k), 7.30-7.81 ppm (-C***H***- of Fmoc).

^{1}H NMR spectrum of NH_2-PEG$_{2k}$-DSPE in $CDCl_3$ was shown in **Figure S2c**. The characteristic peaks of NH_2-PEG$_{2k}$-DSPE matched well with the expected chemical shifts as follow: ^{1}H NMR (400 MHz, $CDCl_3$, 298 K.): δ (ppm) = 0.89 ppm ($C\boldsymbol{H_3}CH_2(CH_2CH_2)_7CH_2COO$- of DSPE, a), 1.27 ppm ($CH_3C\boldsymbol{H_2}(C\boldsymbol{H_2}C\boldsymbol{H_2})_7CH_2COO$- of DSPE, b), 1.60 ppm (-$OCH_2C\boldsymbol{H_2}NHCO$- of DSPE, c), 2.31 ppm (-$OC\boldsymbol{H_2}CH_2NHCO$- of DSPE, d), 3.09 ppm ($CH_3CH_2(CH_2CH_2)_7C\boldsymbol{H_2}COO$- of DSPE, e), 3.65 ppm (-NHCO-$(C\boldsymbol{H_2}C\boldsymbol{H_2}O)_{45}$- of PEG, f), 4.21 ppm (-$COOCH_2CH(OCOCH_2$-$)C\boldsymbol{H_2}O$- of DSPE, g), 4.39 ppm (-$COOC\boldsymbol{H_2}CH(OCOCH_2$-$)CH_2O$- of DSPE, h), 5.25 ppm (-$COOCH_2C\boldsymbol{H}(OCOCH_2$-$)CH_2O$- of DSPE, i).

^{1}H NMR spectrum of DBCO-PEG$_{2k}$-DSPE in $CDCl_3$ was shown in **Figure S2d**. The

characteristic peaks of DBCO-PEG$_{2k}$-DSPE matched well with the expected chemical shifts as follow: ^{1}H NMR (400 MHz, $CDCl_3$, 298 K.): δ (ppm) = 0.88 ppm (C***H$_3$***$CH_2(CH_2CH_2)_7CH_2$COO- of DSPE, a), 1.26 ppm (CH_3C***H$_2$***(C***H$_2$***C***H$_2$***)$_7$$CH_2$COO- of DSPE, b), 1.59 ppm (-OCH_2C***H$_2$***NHCO- of DSPE, c), 2.28 ppm (-OC***H$_2$***CH_2NHCO- of DSPE, d), 3.09 ppm ($CH_3CH_2(CH_2CH_2)_7$C***H$_2$***COO- of DSPE, e), 3.64 ppm (-NHCO-(C***H$_2$***C***H$_2$***O)$_{45}$- of PEG, f), 4.22 ppm (-COOCH_2CH(OCOCH_2-)C***H$_2$***O- of DSPE, g), 4.37 ppm (-COOC***H$_2$***CH(OCOCH_2-)CH_2O- of DSPE, h), 5.22 ppm (-COOCH_2C***H***(OCOCH_2-)CH_2O- of DSPE, i), 7.34-7.74 ppm (-C***H***- of DBCO).

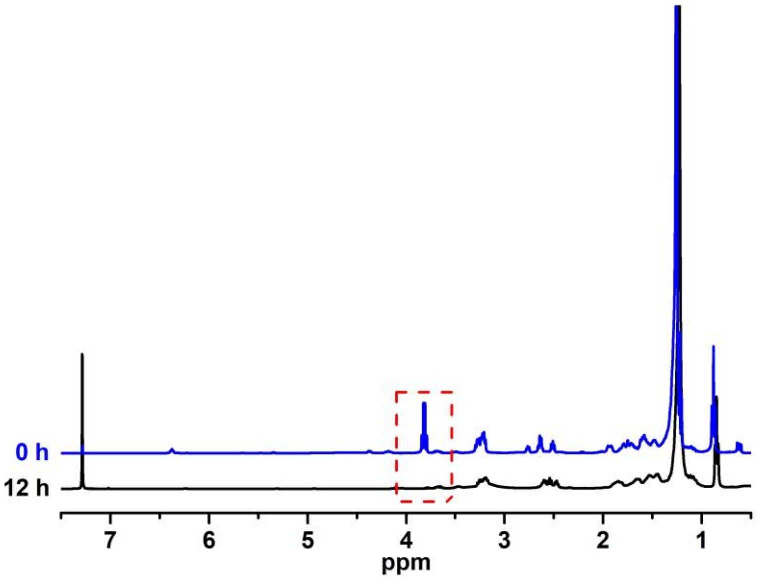

Figure S3. ^{1}H NMR spectrum of Si-lipid in $CDCl_3$ before (0 h) or after incubation in aqueous solution of pH 4 overnight (12 h). Red rectangle indicated that the methylene protons in the ethoxy group of the Si-lipid (($CH_3C\boldsymbol{H_2}O)_3Si$-) almost disappeared after 12 h incubation.

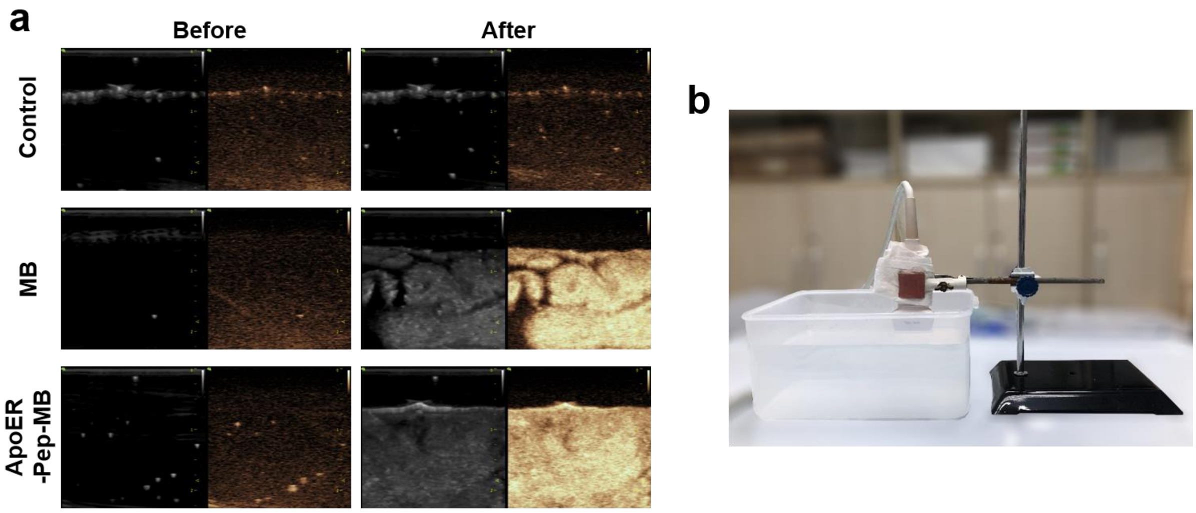

Figure S4. CEUS imaging of MB and ApoER-Pep-MB. (**a**) CEUS imaging of water before and after adding MB or ApoER-Pep-MB (mechanical index, 0.04; frequency, 4.5 MHz; dynamic range, 80 dB). Left: gray scale; right: harmonic in each pair of images. (**b**) Microbubbles were added about 2 cm away from the transducer below water, and the focal zone was adjusted to the 1.8 cm deep location.

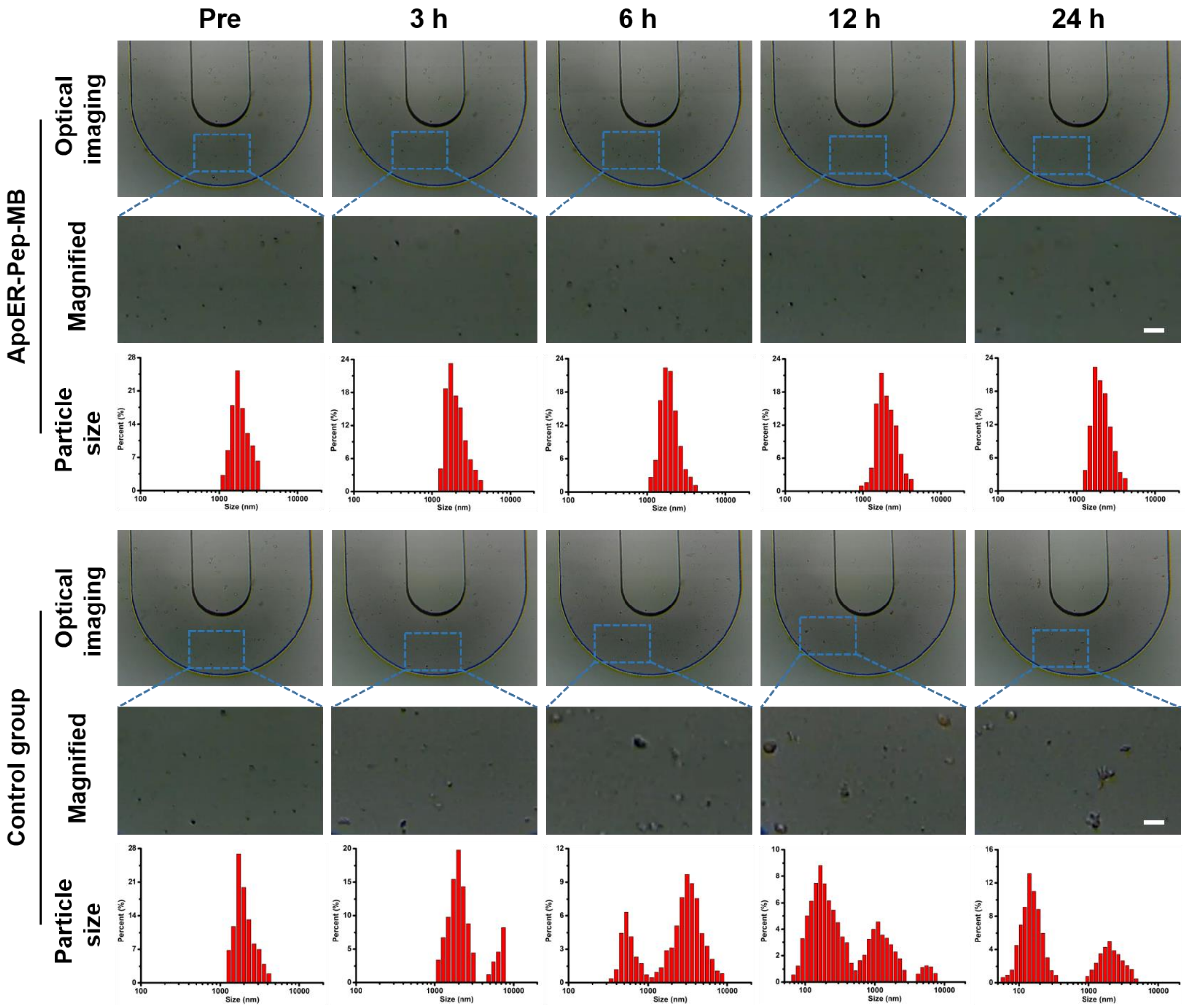

Figure S5. Colloidal stability of microbubbles detected by microfluidic chip. The solution of microbubble with (ApoER-Pep-MB) or without adding Si-lipid (control) was allowed to flow through the channels and then measured by DLS (scale bar: 10 μm).

Table S1. Comparison of perfusion parameters from CEUS imaging in brains of mice injected with MB or ApoER-Pep-MB.

Parameter	MB	ApoER-Pep-MB
Time to peak (TTP, s)	7.98	6.66
Peak intensity (PI, a.u.)	120.71	122.11
Slope coefficient of the WI (a.u./s)	40.71	56.98
Slope coefficient of the WO (a.u./s)	-0.25	-0.04
Time from peak to half (TTH, s)	132.45	463.35

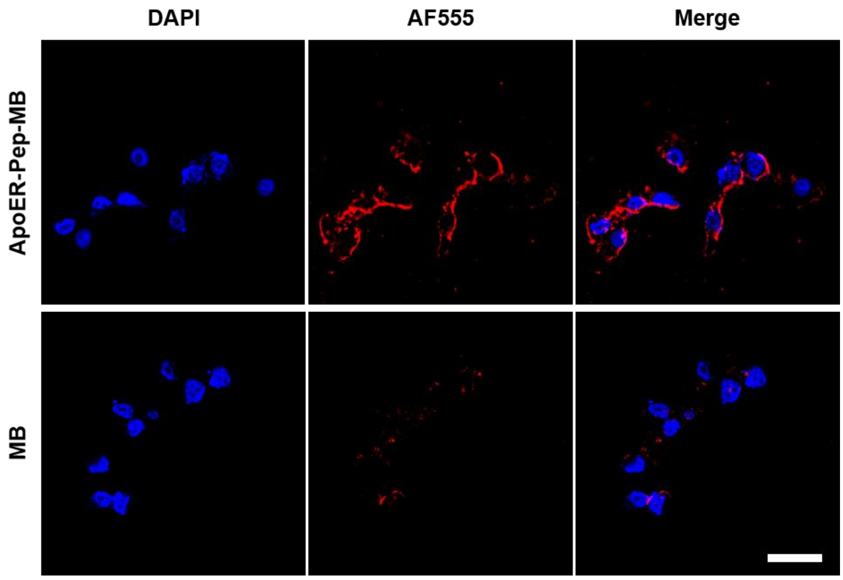

Figure S6. CLSM images of bEnd3 cells after incubation for 10 min with ApoER-Pep-MB@AF555 or MB@AF555 (scale bar: 50 μm).

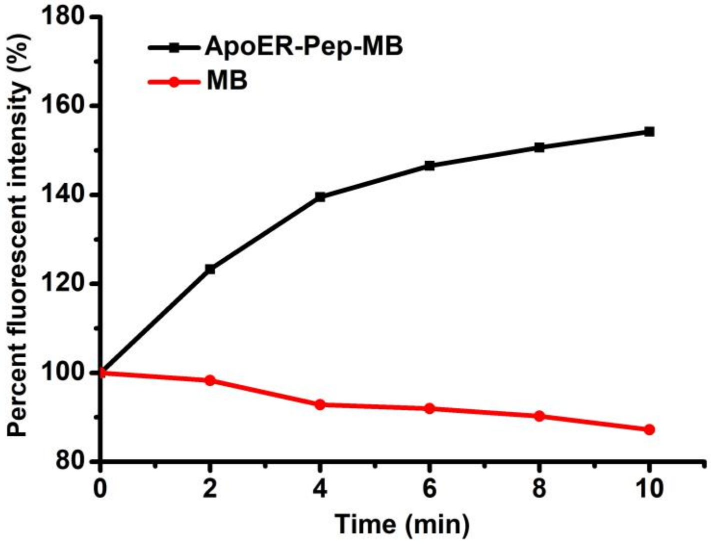

Figure S7. Plotting of normalized mean fluorescence intensities in Figure 2g against time using Image J.

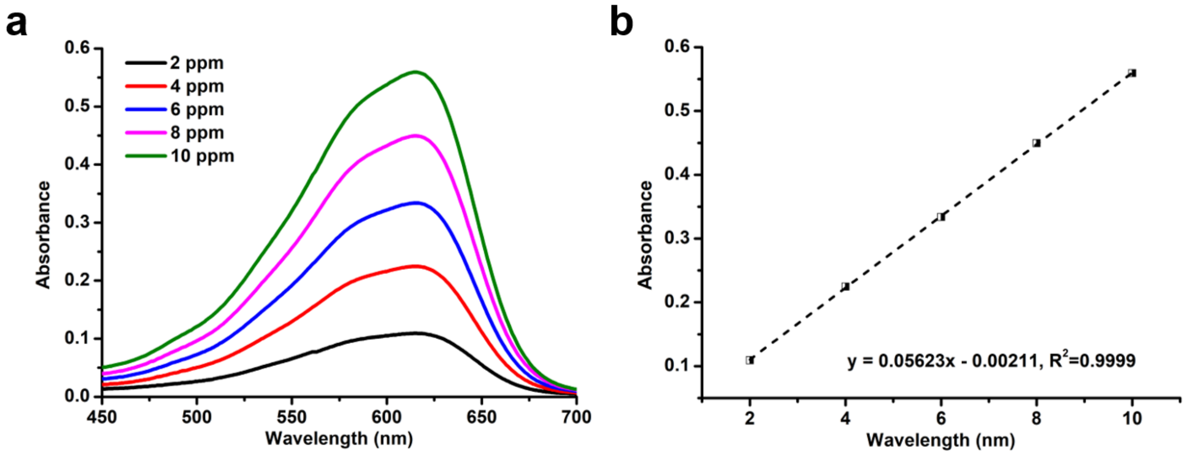

Figure S8. Determination of EB content. (**a**) UV-vis absorbance of EB in 50% trichloroacetic acid solution at various concentrations. (**b**) Standard curve for measuring EB content in 50% trichloroacetic acid solution.

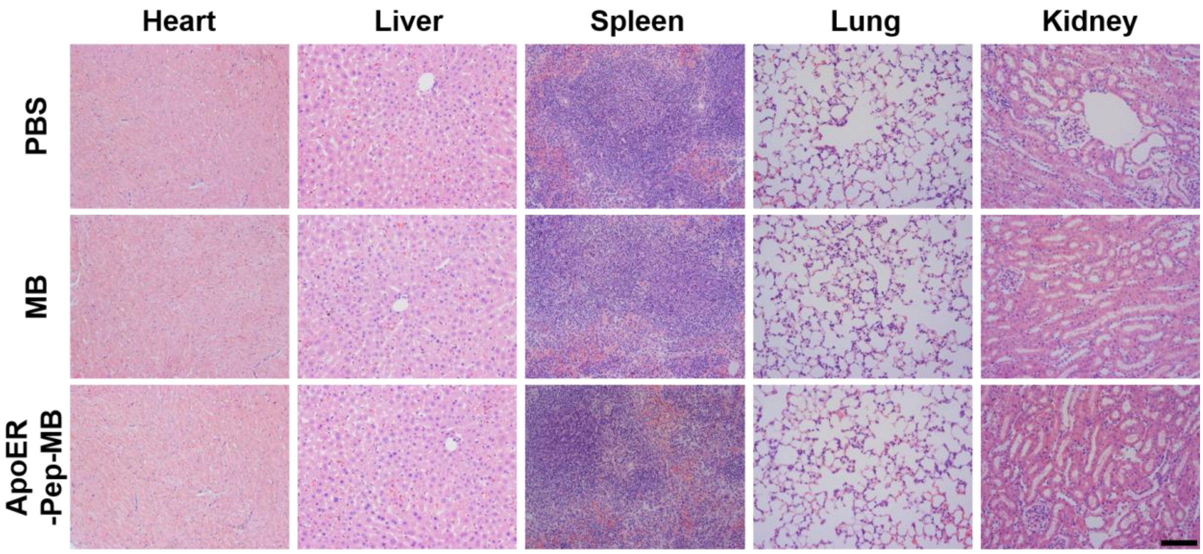

Figure S9. Biosafety assessment of ApoER-Pep-MB and MB applied *in vivo*. H&E staining of various organ tissues from mice receiving different treatments (scale bar: 100 μm).

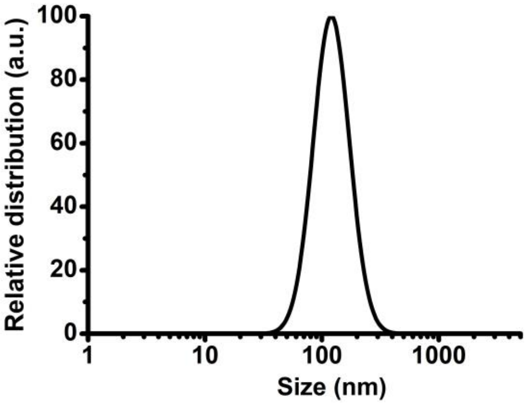

Figure S10. Characterization of Abraxane. Particle size of Abraxane measured by DLS.

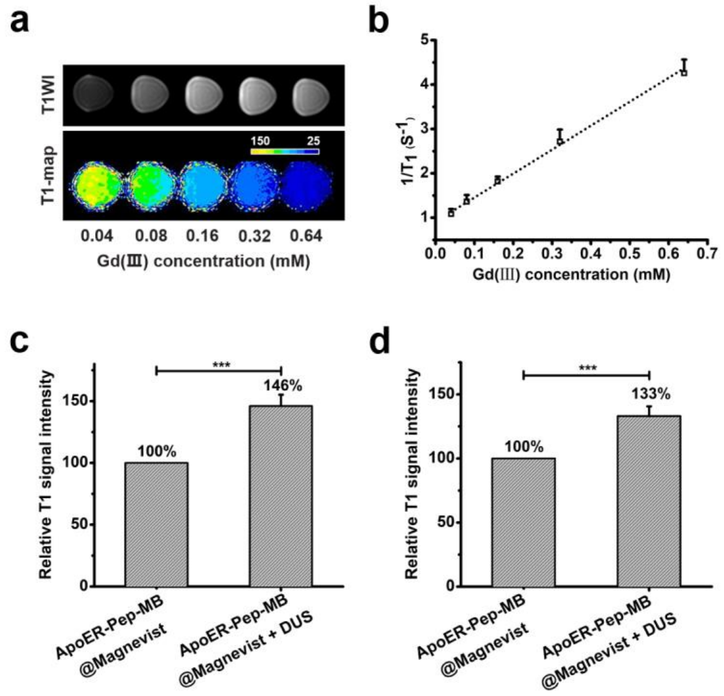

Figure S11. Delivery of Magnevist into brain via DUS-triggered cavitation effect of ApoER-Pep-MB. (**a**) T1-weighted images (T1WI) and T1-map images of Magnevist solution at various Gd concentrations. (**b**) T1 relaxation rates (s^{-1}) as a function of Gd concentration (mM) for Magnevist. The T1 relaxation rates (s^{-1}) were plotted against the Gd concentration to obtain the T1 relaxivity ($r1 = 5.39\ mM^{-1}\ s^{-1}$). (**c**) Mice receiving ApoER-Pep-MB@Magnevist showed a 146% or (**d**) 133% increase in the normalized T1-weighted signal intensity of ultrasound-irradiated area at 0.5 h post-irradiation in the sectional view of transverse plane or coronal plane, respectively. Data are expressed as mean ± SD, n = 3, ***$P < 0.001$.

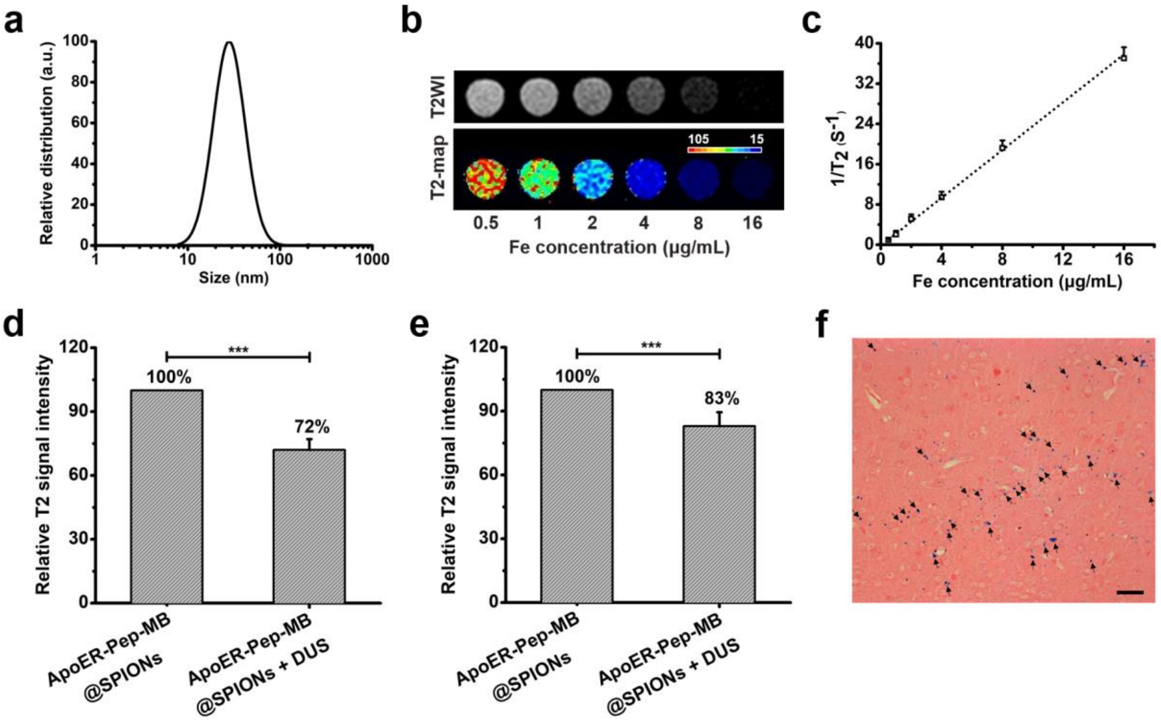

Figure S12. Delivery of water-soluble SPIONs into brain via DUS-triggered cavitation effect of ApoER-Pep-MB. (**a**) Particle size of water-soluble SPIONs. (**b**) T2-weighted (T2WI) and T2-map images of SPIONs solutions at various Fe concentrations. (**c**) T2 relaxation rates (s^{-1}) as a function of Fe concentration (μg mL^{-1}) for SPIONs. The T2 relaxation rates (s^{-1}) were plotted against the Fe concentration to obtain the T2 relaxivity (r2 = 2.37 mL μg^{-1} s^{-1}). (d) Mice receiving ApoER-Pep-MB@SPIONs showed a 72% or (**e**) 83% decrease in the normalized T2-weighted signal intensity of ultrasound-irradiated area at 0.5 h post-irradiation in the sectional view of transverse plane or coronal plane, respectively. Data are expressed as mean ± SD, n = 3, ***$P < 0.001$. (**f**) Prussian blue staining of brain tissue sections from mice after the ApoER-Pep-MB@SPIONs + DUS treatment (scale bar: 50 μm). Black arrows indicate SPIONs stained blue by Prussian blue.

Experimental Procedures

Materials.

ApoER(159-167)$_2$ peptide with a 6-azido-hexanoic acid on N-terminal (ApoER-Pep-N$_3$, sequence: (LRKLRKRLL)$_2$C-N$_3$, 95%) was obtained from Wuhan Holder Co., Ltd. (Wuhan, China). Hexadecylamine, bromohexadecane, and 3-aminopropyltriethoxysilane were purchased from Aladdin Industrial Corporation (Shanghai, China) and used as received. 1,2-Distearoyl-snglycero-3-phosphatidylcholine (DSPC), indocyanine green (ICG), Evans Blue (EB) and dihexadecyl phosphate were purchased from TCI Development Co., Ltd. (Shanghai, China). 1,1'-Dioctadecyl-3,3,3',3'-tetramethylindocarbocyanine perchlorate (DiI iodide), succinic anhydride, dry tetrahydrofuran, citric acid, dicyclohexylcarbodiimide (DCC), and dry dichloromethane were purchased from J&K Scientific Ltd. (Beijing, China). Sodium dicetylphosphate (Dicet), 1, 2-distearoyl-sn-glycero-3-phosphoethanolamine-N-[methoxy (polyethylene glycol)-2000] (PEG$_{2k}$-DSPE), dibenzocyclooctyne acid (DBCO acid), 1,2-distearoyl-sn-glycero-3-phosphoethanolamine (DSPE) and Fmoc-NH-(polyethylene glycol)-2000 acetic acid (Fmoc-PEG$_{2k}$-COOH) were purchased from Guangzhou Tanshui Technology Co., Ltd. Dialysis bag was purchased from Green Bird Technology Development Co., Ltd. (Shanghai, China). Na_2CO_3 solid, $MgSO_4$ solid, NaCl solid, anhydrous ethanol, chloroform, hexane, acetonitrile, ethyl acetate, glycerol and propylene glycol were purchased from Chemical Reagent Factory (Guangzhou, China). Magnevist, Albumin Bound, and SonoVue® were kindly provided by the Third Affiliated Hospital of Sun Yat-sen University.

The anti-Iba1 antibody (rabbit anti-mouse) (ab178846), anti-GFAP antibody (rabbit anti-mouse) (ab7260), Alexa Fluor® 488-labeled goat anti-rabbit IgG (ab150077), goat anti-rabbit IgG antibody (HRP) (ab205718) and DAB Substrate Kit (ab64238) were purchased from Abcam Inc. (Cam-bridge, MA, USA). The anti-Occludin antibody (rabbit anti-mouse) (27260-1-AP), ZO-1 antibody (rabbit anti-mouse) (21773-1-AP) were purchased from Proteintech group (Wuhan, China). In Situ Cell Death Detection Kit and POD (11684817910) was purchased from Roche Diagnostics GmbH (Penzberg, Germany). Fluorescein isothiocyanate (FITC)-conjugated lectin (L0401) was purchased from Sigma-Aldrich (Prague, Czech Republic). Alexa Fluor® 555 (AF555)-labeled mouse IgG was produced from Bioss (Beijing, China). All other reagents used in this study were commercially available and of analytical purity grade or higher.

Synthesis of N-[N-(3-Triethoxysilyl) propylsuccinamoyl] dihexadecylamine (Si-lipid).

The synthetic approach to the lipidic organoalkoxysilane (Si-lipid) with a molecular structure analogous to lipids was shown in **Figure S1a** by referring to a previous report with minor modifications.[1] First, the molecule dihexadecylamine was obtained by a combination reaction. Briefly, 6.18 g of hexadecylamine (25.59 mmol), 6.50 g of bromohexadecane (21.29 mmol), and 5.66 g of Na_2CO_3 were placed in a 100 mL Schlenk flask, after adding 50 mL of anhydrous ethanol, the mixture was heated to reflux for 5 days. Afterwards, the solvent was removed by rotary evaporation at room temperature and the residual solid was suspended in $CHCl_3$. The mixture was washed with water, then dried over anhydrous $MgSO_4$. After removing the solvent by evaporation, the residue was recrystallized from hexane for 3 times, and dried to give a white solid (6.44 g, 13.85 mmol, 65% yield).

Next, 6.00 g of dihexadecylamine (12.89 mmol) and 2.59 g of succinic anhydride (25.78 mmol) were placed in a 200 mL Schlenk flask, after adding 100 mL of dry tetrahydrofuran under an argon atmosphere, the solution was allowed to stir for 24 h at room temperature. The solvent was removed by rotary evaporation and the residual solid was suspended in 50 mL of CH_2Cl_2. The mixture was washed with 10 % aqueous citric acid and saturated aqueous NaCl successively, then evaporated in vacuo. The N, N'-dihexadecylsuccinamic acid was obtained by recrystallization from acetonitrile as a white solid (4.96 g, 8.77 mmol, 68% yield).

Finally, 2.00 g of DCC (9.69 mmol) and 4.75 g of N, N'-dihexadecylsuccinamic (8.40 mmol) were dissolved in 100 mL of dry dichloromethane at 0℃ with stirring for 15 min. Then 2.42 g of 3-aminopropyltriethoxysilane (10.92 mmol) was added and the mixture was kept stirring for 4 h at 0℃ and subsequently turned to room temperature for a further 12 h. Filtration was carried out to remove precipitates (N, N'-dicyclohexylurea), the solution was then evaporated in vacuo and the residual oil was purified by column chromatography. Impurities were eluted with ethyl acetate/chloroform (v/v = 1:9); subsequent elution with 100% ethyl acetate gave a colorless oil (2.91 g, 3.78 mmol, 45% yield), TLC: Rf=0.26 (chloroform/ethyl acetate = 20:1).

Synthesis of Dibenzocyclooctyne-conjugated 1,2-distearoyl-sn-glycero-3-phosphoethanolamine-N-[methoxy (polyethylene glycol)-2000] (DBCO-PEG$_{2k}$-DSPE).

0.311 g of Fmoc-PEG$_{2k}$-COOH (0.14 mmol), 0.03 g of NHS (0.28 mmol, 2 eq), 0.05 g of EDC•HCl (0.28 mmol, 2 eq) were dissolved in 13 mL of CH_2Cl_2 in a 50 mL Schlenk flask and kept stirring for 24 h. After adding 7 mL of methanol (MeOH), 1.5 mL of water and 0.10 g of DSPE (0.14 mmol), the reaction was allowed to proceed for a further 24 h at ambient temperature. Afterwards, the solution was dialyzed (MW cut-off: 1000 Da) against methanol

for 48 h, precipitated into diethyl ether, filtered and washed, then dried under vacuum to yield light yellow powder Fmoc-PEG_{2k}-DSPE (0.39 g, 0.13 mmol, 93% yield).

Then, the protective group of Fmoc-PEG_{2k}-DSPE was removed. In brief, 0.33 g of Fmoc-PEG_{2k}-DSPE (0.11 mmol) was dissolved in 16 mL of $CHCl_3$, followed by the addition of 4 mL of piperidine while maintaining a piperidine concentration of approximately 20% throughout the solution. After stirring for half an hour, the solution was dialyzed (MW cut-off: 1000 Da) against methanol for 24 h, precipitated into diethyl ether, filtered and washed, then dried under vacuum to yield white powder NH_2-PEG_{2k}-DSPE (0.25 g, 0.09 mmol, 81% yield).

Next, 0.04 g of DBCO acid (0.14 mmol), 0.03 g of NHS (0.28 mmol, 2 eq), 0.05 g of EDC•HCl (0.28 mmol, 2 eq) were dissolved in 10 mL of CH_2Cl_2 in a 25 mL Schlenk flask. After stirring for 24 h, 0.38 g of NH_2-PEG_{2k}-DSPE (0.14 mmol) was added. The mixture was stirred for a further 24 h, then dialyzed (MW cut-off: 1000 Da) against methanol for 24 h, precipitated into diethyl ether, filtered and washed, dried under vacuum to yield white powder DBCO-PEG_{2k}-DSPE (0.36 g, 0.12 mmol, 86% yield).

Preparation of microbubbles.

The thin-film hydration method was carried out to prepare various microbubbles used in this experiment. In brief, a 5:1:4:2 molar ratio of DSPC: DBCO-PEG_{2k}-DSPE: Si-lipid: Dicet (a total of 32 mg) was dissolved in 5 mL of chloroform to prepare the BCECs-bindable microbubbles ApoER-Pep-MB. The solvent was removed by rotary evaporation at 37℃ until a thin lipid film formed. Next, the film was hydrated by vortex mixing for 5 min with a 5mL solution of 8:1:1 (v/v/v) water: glycerol: propylene glycol at 60°C. Then 2 mg of ApoER-Pep-N_3 was added and the reaction was allowed to proceed for 2 h. Next, the pH value of the solution was adjusted to pH 4 and incubated overnight to allow siloxane networks developing on the surfaces of the microbubbles.[1] The solution was dialyzed (MWCO: 2000 kDa) against 8:1:1 (v/v/v) water: glycerol: propylene glycol for 1 d to remove small aggregates and concentrated to 5 mL using a Millipore Centrifugal Filter Device (MW cut-off: 100 kDa). Afterwards, the solution was sub-packaged into 10 vials (0.5 ml each vial). Gas in each vial was replaced with perfluoropropane gas (C_3F_8) and the solution was mechanically agitated for 45 s using a VialMix shaker (YG-100, ZOGEAR, Shanghai, China) to form BCECs-bindable microbubbles (ApoER-Pep-MB) with a siloxane shell and a C_3F_8 gas core. The non-bindable microbubbles (MB) were treated likewise except for adding the binding peptide ApoER-Pep-

N_3. The concentration of microbubbles was determined under microscope by a hemocytometer.

Characterization of polymers and microbubbles.

^{1}H NMR spectra were recorded on a Varian Unity 400 MHz Spectrometer (Bruker, Switzerland) in various deuterated solvents. Fourier transform infrared (FTIR) spectral measurements were performed using a Nicolet/Nexus 670 FTIR spectrometer (Thermo Fisher Scientific, USA) with a resolution of 2 cm^{-1} and the powder samples were compressed into KBr pellets. The size and zeta potential were determined by dynamic light scattering (DLS) on a 90 Plus/BI-MAS instrument (Brookhaven Instruments Corporation, USA). Transmission electron microscopy (TEM) images were obtained from a model H-7650 TEM (Hitachi Ltd, Tokyo, Japan) operated at 120 kV. The morphology of microbubbles loaded with DOX was visualized under confocal laser scanning microscopy (LSM 710; Carl Zeiss Microscopy GmbH, Jena, Germany). The content of TMZ or EB was measured using a Unico UV-2000 UV-vis spectrophotometer (Shanghai, China) based on the standard absorption curve of TMZ or EB absorption.

Furthermore, the gadolinium (Gd) or iron (Fe) content was determined by an Optima 5300 DV ICP-AES (Perkin Elmer® Inc., USA) or a polarized Zeeman Atomic Absorption Spectrometer (AAS) (Model: Z-2000 series), respectively. The Gd concentration was measured at the wavelength of 342 nm, and the Fe concentration was measured at the specific absorption wavelength of 248.3 nm. The MRI sensitivity of Magnevist or SPIONs was evaluated by measuring the T1 or T2 relaxation time on a clinical MR system (Ingenia 3.0T; Philips Medical Systems, Best, Netherland), respectively. To obtain the T1 relaxivities, Magnevist was dissolved in PBS at designed concentrations in 96-well detachable plate. An inversion recovery spin echo sequence was performed with the following parameters: TR = 1500 ms; TE = 20 ms; FOV, 80 mm × 80 mm; matrix, 228 mm × 289 mm; voxel, 0.4 mm × 0.4 mm; slice thickness, 2 mm; reconstruction matrix, 512; IR delay, 400 ms; NSA, 1. Regions of interest (ROI, mean size, 30 mm^2) were checked to obtain the T1 relaxation times. And the T1 relaxivity value (r_1) was calculated from the slope of the linear plots of 1/T1 versus Gd concentration determined by linear least-squares regression analysis. For the T2 relaxivities, SPIONs were treated likewise. The multi-echo T2-mapping sequence was performed with the following parameters: TR = 1800 ms; TE = 13 ms; FOV, 120 mm × 120 mm; matrix, 256 × 256; voxel, 0.47 mm × 0.47 mm; slice thickness, 2 mm; flip angle, 90°; NSA, 2. The regions of interest (mean size, 30 mm^2) were checked to obtain the T2 relaxation

times. And the T2 relaxivity value (r_2) was calculated from the slope of the linear plots of 1/T2 versus Fe concentration determined by linear least-squares regression analysis. The method of MRI sensitivity measurements was described in detail in our previous report.[2]

Afterwards, to assess the imaging ability of ApoER-Pep-MB and MB, contrast-enhanced ultrasound (CUES) imaging experiment was carried out with a high-frequency linear array transducer X4-12L (VINNO 70, China). 20 μL of ApoER-Pep-MB or MB (2.5×10^4 microbubbles/mL) was respectively added to a transparent box filled with 1 L of double distilled water. CEUS imaging was performed immediately after the injection of microbubbles about 2 cm away from the transducer hanging in water (**Figure S4b**). The focal zone was placed at 1.8 cm in depth, and the imaging parameters are listed as follows: mechanical index of 0.04, frequency of 4.5 MHz, dynamic range of 80 dB.

Besides, to verify that Si-lipid could be hydrolyzed under the experimental conditions, Si-lipid was assembled into particles by ultrasound and incubated in aqueous solution of pH 4 overnight (for about 12 h). Then, Si-lipid was dialyzed against water, freeze-dried, and dissolved in $CDCl_3$ to detect its structure by ^{1}H NMR. The colloidal stability of microbubbles was further detected by the microfluidic chip equipped with a peristaltic pump according to a previous study[3]. The solution of microbubble with (ApoER-Pep-MB) or without adding Si-lipid (control) was allowed to flow through the channels. The microfluidic chip was observed under a microscope. At pre-designed time points, the solution flowing through the channel was photographed and collected, then measured by DLS to characterize its particle size.

Finally, to longitudinally compare the stability of ApoER-Pep-MB and MB *in vitro*, a CEUS imaging experiment was performed on a VisualSonics Vevo 3100 Imaging System (VisualSonics, Canada) with an ultra-high frequency linear array transducer MX 250. The imaging parameters were listed as follows: frequency of 18 MHz; transmitted power of 10%; contrast gain of 30.0 dB. 1.5 ml of ApoER-Pep-MB or MB with a concentration of 2.5×10^4 microbubbles/mL were added to the respective sample wells of a custom-made 2% (w/v) agarose phantom.[4] The focal zone was placed at the center of wells, and acquisitions were carried out at a rate of 25 frames per second every 30 minutes.

Evaluation of microbubble binding to BBB *in vivo*

Animal models. Eight-week-old BALB/c nude mice were provided by Beijing Vital River Laboratory Animal Technology Co. Ltd. (Beijing, China) and carefully housed in specific pathogen-free (SPF) environment with access to water and food ad libitum on a 12 h light-dark cycle.

***In vivo* fluorescence imaging.** *In vivo* fluorescence imaging studies were first performed to investigate the biodistribution of microbubbles in nude mice of eight weeks old. Images were acquired on mice with a small animal *in vivo* fluorescence imaging system (In-Vivo Imaging System FX Pro, Carestream Health Inc., New Haven, CT, USA), the excitation and emission wavelengths of ICG were 720 and 790 nm, respectively. Next, mice (n = 3) were tail vein injected with indocyanine green (ICG)-loaded ApoER-Pep-MB or MB (500 μg/kg ICG). 4 min later, the mice in MB + DUS group and ApoER-Pep-MB + DUS group received a treatment by DUS. All mice were scanned again by the *in vivo* fluorescence imaging system 30 min post-sonication. Afterwards, the mice were sacrificed, and the major organs (heart, liver, spleen, lungs and kidneys) and brains were excised for *ex vivo* fluorescence imaging.

Quantitative analysis of contrast-enhanced ultrasound (CEUS) images. The binding efficiency of microbubbles was then evaluated with the time-intensity method under continuous B-mode and CEUS imaging. Mice with their heads restrained were anesthetized by injection of 3% pentobarbital sodium (2.5 mL/kg) intraperitoneally. After applying a small amount of coupling gel to the scalp, continuous real-time ultrasonographic imaging was performed on a LOGIQ E9 system (General Electric, USA) with a linear transducer 9L (General Electric, USA) in both conventional and contrast mode. Acquisitions started after injecting 50 μL of MB (2.5×10^6 microbubbles/mL) into mice through the tail vein and performed with a duration time of 30 min. After complete clearance of MB, continuous B-mode and CEUS imaging was carried out on the same mice injected with 50 μL of ApoER-Pep-MB (2.5×10^6 microbubbles/mL). Images of CEUS were analyzed with Sonomath software (AmbitionT.C., Chongqing, China) offline. The ROIs were kept constant for the same mice during imaging. Echo power intensities within the ROIs were automatically calculated. A raw time-intensity curve was plotted. Then a mathematic equation model was used to fit the raw time-intensity curves. Afterwards, the raw signal data was fitted into the mathematical model developed at the Gustave Roussy Institute.[5]

$$I(t) = a_0 + (a_1 - a_0) * \left[\frac{A + \left(\frac{t}{a_2}\right)^p}{B + \left(\frac{t}{a_2}\right)^q} \right]$$

In this model, I(t) describes the variation in the intensity of contrast uptake as a function of time; a_0 is the intensity before the arrival of the contrast agent; a_1 is linked to the maximum value of contrast uptake; a_2 is linked to the rise time to the peak intensity; p is a coefficient related to the increase in intensity; q is a coefficient related to the decrease in intensity; A and B are arbitrary parameters. Data from the fitted curve was analyzed with Origin (OriginLab,

USA) to generate the following semi-quantitative parameters: Peak intensity (PI), Time to peak (TTP), Slope coefficient of the wash in (WI), Slope coefficient of the wash out (WO), Time from peak to half (TTH) according to a previous literature.[6]

***In vitro* CLSM observation.** Mouse brain microvascular endothelial cells (bEnd3) were purchased from BeNa Culture Collection Biotechnology Co., LTD (Beijing, China), and cultured at 37 °C in DMEM medium supplemented with 10% FBS and 1% penicillin-streptomycin in a humidified incubator of 5% CO_2. The bEnd3 cells were seeded at a density of 1.0×10^6 cells per well into a 12-well plate and cultured at 37 °C overnight. After incubated with ApoER-Pep-MB@AF555 or MB@AF555 for 10 min, the cells were washed with fresh PBS and stained with DAPI, then observed under CLSM.

Intravital real-time observation by two-photon CLSM. The real-time two-photon CLSM (Olympus FVMPE-RS, Japan) was used to directly observe the microbubbles binding to the cerebral vessels of nude mice. ApoER-Pep-MB and MB were stained by DiI to make themselves visible under CLSM. Mice (n = 3) with their heads restrained were anesthetized by the injection of 3% pentobarbital sodium (2.5 mL/kg) intraperitoneally. Then, a hand-held cranial drill (RWD Life Science, China) was used to polish a small skull region (~4 mm in diameter) under a dissection microscope.[7] The mice were then injected intravenously with FluoSpheres™ carboxylate-modified microspheres to mark the blood vessel. Further, mice were injected with DiI-loaded ApoER-Pep-MB or MB (500 μg/kg DiI) and dripped with a drop of water on the thinned-skull cranial window. Afterwards, the mice were slightly placed on a thermoplate of the CLSM, and the height of the objective lens was exactly adjusted to touch the water droplet. The experiment was carried out by an Olympus two-photon system, a pre-programmed program was executed every 2 min, taking 30 images in succession once time. The entire procedure was cycled 6 times for a total time of 12 min. Images were obtained by Olympus FV1000 software and analyzed using Image J.

BBB opening and recovery.

The BBB was opened by a high-frequency linear array transducer X4-12L (VINNO 70, China) with a frequency of 4 MHz, and EB was chosen as an indicator to evaluate the difference in the BBB permeability of mice treated with ApoER-Pep-MB, MB, or SonoVue® under DUS irradiation. Mice with their heads immobilized were anesthetized intraperitoneally by the injection of 3% pentobarbital sodium (2.5 mL/kg). Ultrasound transmission gel was applied between the scalp of mice and the transducer hanging above the parietal bone to maximize transmission of the ultrasound. Sonication was applied at the middle of brain along

the rostral-caudal axis with the aid of a stereotaxic apparatus and real-time guidance of CEUS imaging. 4 min after injecting 50 μL of ApoER-Pep-MB, MB, or SonoVue® (2.5×10^6 microbubbles/mL) through the tail vein, sonication was performed for 3 min using the following parameters, which was described according to previous literature[8]: 22.5 cycles of wave formed a single ultrasound pulse, which generated a tone-burst at an ultrasound fundamental frequency of 3 MHz for a tone-burst duration of 7.5 μs. Such ultrasound pulses were repeated at a pulse repetition frequency of 40 Hz within the ultrasound duration of 0.6 s. The inter-sonication interval was 2 s. The mechanical index was 0.3. The acoustic power was calculated to be 0.52 MPa. 0.5 h later, the mice were perfused with PBS, and their brains were ground to fully extract EB with 50% trichloroacetic acid solution.

Afterwards, the position of the transducer was changed to verify whether the BBB could be opened in any position with assistance of DUS-mediated ApoER-Pep-MB. The experimental procedure was carried out likewise. In brief, mice were injected with 50 μL of ApoER-Pep-MB (2.5×10^6 microbubbles/mL) through the tail vein, sonication was performed for 3 min using the above parameters 4 min after injection. Then mice were perfused with 4% paraformaldehyde solution. The brains were collected and sectioned coronally into 1.5 mm sections.

Finally, to evaluate the duration of BBB opening assisted by ApoER-Pep-MB, EB was intravenously injected at various preset time points (0 h, 0.5 h, 1 h, 2 h) after sonication. Mice were perfused with PBS, and their brains were collected. EB was extracted with 50% trichloroacetic acid solution and then measured by UV-vis to evaluate the permeability of the BBB.

Western blot analysis and Co-Immunoprecipitation (Co-IP) assay

Brain tissues were homogenized in an ice-cold RIPA buffer (for western blot analysis) or Immunoprecipitation buffer (for Co-IP assay). The homogenate was centrifuged at 15,000 g for 30 min. The protein content in the supernatant was determined using a NanoDrop 1000 spectrophotometer (ThermoFisher Scientific, USA). Then, western blot analysis was carried out.

For Co-IP assay, supernatants containing 1 mg of total protein in 500 μL of IP buffer were first precleared with protein A Sepharose beads (GE Health care) for 1 h. Proteins were then incubated with anti-ZO-1 (1:500) or anti-Occludin (1:500) antibody overnight at 4 ℃. A non-specific IgG antibody was used as negative control in the reaction. The samples were further incubated with protein A Sepharose beads for 4 h at 4°C. The formed immunocomplexes were

centrifugated at 250 g for 1 min. The precipitates were collected and washed three times with IP buffer, then mixed with 3× SDS sample buffer, boiled for 5 min and analyzed with western blotting. Input proteins (supernatants containing total protein) were used as positive control in western blotting.

Transmission electron microscopy (TEM) observation

Mice were injected with 50 μL of ApoER-Pep-MB or MB (2.5×10^6 microbubbles/mL) through the tail vein, sonication was performed 4 min after injection. Afterwards, mice were transcardially perfused with 0.1 M PBS, followed by 2.5% glutaraldehyde. The brain tissue of mice was collected and post-fixed in 2.5% glutaraldehyde, followed by 1% osmium tetroxide. Tissue blocks were dehydrated and embedded in epoxy resin. Sections were cut with a diamond knife on a LeicaEMUC7 ultramicrotome (Leica Microsystems) at a thickness of 100 nm and then observed by a Hitachi model H-7650 TEM operated at 120 kV.

Biosafety assessment

Immunofluorescence staining. Mice (n = 3) were perfused with ice-cold PBS and 4% paraformaldehyde (PFA) in deep anesthesia after DUS treatment, followed by their brains dissected, frozen and sliced coronally into 20 μm sections. Further, antigen retrieval was carried out with citric acid (pH 6.0) at 95°C for 15 min. The sections were permeabilized with 0.3% triton X-100 at room temperature for 10 min, then blocked by goat serum for 1 h. Afterwards, the sections were incubated with rabbit anti-Iba1 or rabbit anti-GFAP for 16 h at 4°C, washed with TRIS-buffered saline (TBS), sequentially incubated with goat anti-rabbit IgG Alexa Fluor® 488 antibody (8 μg/mL) for 0.5 h at room temperature and finally counterstained with DAPI (Solarbio, China) for observation using a fluorescence microscope (Leica DM2000, Germany).

TUNEL assay. 5 μm paraffin sections were obtained from murine brains by the same procedure of immunohistochemical staining. TUNEL assay was carried out using an *In Situ* Cell Death Detection Kit according to the manufacturer's protocol. After counterstaining with DAPI, images were acquired with Pannoramic MIDI (3DHISTECH, Hungary).

Histologic Analysis. Paraffin sections of murine brains were obtained as mentioned above. Sections were stained with hematoxylin and eosin or methylene blue according to standard protocol. Images were acquired with Hamamatsu (NanoZoomer S360, Japan) and analyzed with NanoZoomer Digital Pathology (NanoZoomer S360, Japan).

Varisized therapeutic or diagnostic agents transport into brain.

Indocyanine green (ICG). An *in vivo* photoacoustic (PA) imaging on LOIS-3D Pre-clinical Mouse Imaging System (Tomowave, USA) with an excitation wavelength of 755 nm was used to monitor the extravasation of ICG. A brain-focused PA imaging was carried out before and after 50 μL of ICG-loaded MB or ICG-loaded ApoER-Pep-MB (2.5×10^6 microbubbles/mL) injected into mice through the tail vein. Sonication was applied according to the procedure mentioned above at 4 min after the injection of various kinds of microbubbles. And the brain-focused PA imaging was performed again after 0.5 h. Procedures for the three-dimensional PA tomographic reconstruction were described in detail by previous work.[9]

Antibody. AF555-labeled IgG (Bioss, China) was employed as an example to verify whether antibodies could enter the brain with assistance of DUS-mediated ApoER-Pep-MB. Mice were injected with 50 μL of IgG-encapsulated ApoER-Pep-MB (2.5×10^6 microbubbles/mL) through the tail vein and treated as described above. In 0.5 h post-sonication, FITC-labeled lycopersicon esculentum lectin (FITC-Lectin, 0.05 mg per mouse) was injected intravenously. Then, mice were sacrificed after 5 min and brains were dissected, frozen, sliced into 20 μm sections and counterstained with DAPI. Photographs were taken with a CLSM and images were processed with NIS-Elements.

Abraxane. To determine the extravasation of Abraxane, Cy3-labeled Albumin Bound (Bioss, China) was encapsulated in ApoER-Pep-MB and intravenously injected into mice (50 μL, 2.5×10^6 microbubbles/mL), followed by DUS irradiation as described above. Afterwards, FITC-labeled lycopersicon esculentum lectin (FITC-Lectin, 0.05 mg per mouse) was injected through the tail vein in 0.5 h after sonication. 5 min later, mice were sacrificed and the brains were dissected, frozen and sliced into 20 μm sections. Photographs were taken with a CLSM and images were processed with NIS-Elements.

Magnevist. MRI scanning was carried out to further evaluate the ability of ApoER-Pep-MB to transport MR contrast agents into brain of nude mice. Mice (n = 3) were first anesthetized by intraperitoneal injection of 3% pentobarbital sodium (2.5 mL/kg), and then scanned on the 3.0 T MR scanner equipped with a 50 mm×65 mm 8-channel phased-array mouse coil (Shanghai Chenguang Medical Technologies Co., Ltd., Shanghai, China). Sonication was performed 4 min after the mice injected with 50 μL of Magnevist-loaded ApoER-Pep-MB (1.5 mg/mL, 2.5×10^6 microbubbles/mL) via tail vein, and T1-weighted MR images were obtained after 0.5 h using a fast spin echo sequence: TR = 400 ms; TE =11 ms; FOV, 60 mm × 60 mm; matrix, 248 mm × 246 mm; slice thickness, 1 mm; flip angle, 90°; NSA, 2. Images were analyzed using Image J software. Finally, the brain of mice was

harvested and dissolved in a mixture of HNO_3 and HCl (v/v =3:1). The Gd content was then measured by ICP-AES.

Superparamagnetic iron oxide nanoparticles (SPIONs). The water-soluble superparamagnetic iron oxide nanoparticles (SPIONs) of 20 nm in diameter were first synthesized by a previous study.[10] Afterwards, mice (n = 3) were scanned using the Ingenia 3.0 T MR scanner with a 50 mm × 65 mm 8-channel phased-array mouse coil after anesthetization with 3% pentobarbital sodium (2.5 mL/kg). Subsequently, sonication was performed 4 min after the mice injected with 50 μL of SPIONs-loaded ApoER-Pep-MB (2.5×10^6 microbubbles/mL) through the tail vein, and the same MRI scan was repeated after 0.5 h. Images of T2WI were acquired using the following parameters: TR/TE, 1800/100 ms; matrix, 252 mm × 252 mm; FOV, 60 mm × 60 mm; flip angle, 90°; slice thickness, 1 mm; NSA, 2. Images were analyzed by Image J. Finally, the brain of mice was harvested and dissolved in a mixture of HNO_3 and HCl (v/v =3:1). The Fe content was then measured by AAS.

References

[1] K. Katagiri, M. Hashizume, K. Ariga, T. Terashima, J. i. Kikuchi, *Chemistry–A European Journal* **2007**, 13, 5272.

[2] a) B. Li, H. Xiao, M. Cai, X. Li, X. Xu, S. Wang, S. Huang, Y. Wang, D. Cheng, P. Pang, *Advanced functional materials* **2020**, 30, 1909117; b) B. Li, M. Cai, L. Lin, W. Sun, Z. Zhou, S. Wang, Y. Wang, K. Zhu, X. Shuai, *Biomaterials science* **2019**, 7, 1529.

[3] J. Wang, Y. Gu, X. Liu, Y. Fan, Y. Zhang, C. Yi, C. Cheng, M. Yang, *International Journal of Molecular Sciences* **2022**, 23, 10885.

[4] T. Yin, P. Wang, R. Zheng, B. Zheng, D. Cheng, X. Zhang, X. Shuai, *International journal of nanomedicine* **2012**, 7, 895.

[5] M. Gauthier, I. Leguerney, J. Thalmensi, M. Chebil, S. Parisot, P. Peronneau, A. Roche, N. Lassau, *World Journal of Radiology* **2011**, 3, 70.

[6] X. Zhou, P. Zhou, Z. Hu, S. M. Tian, Y. Zhao, W. Liu, Q. Jin, *Journal of Ultrasound in Medicine* **2018**.

[7] G. Yang, F. Pan, C. N. Parkhurst, J. Grutzendler, W.-B. Gan, *Nature protocols* **2010**, 5, 201.

[8] K. Yu, X. Niu, E. Krook-Magnuson, B. He, *Nature communications* **2021**, 12, 2519.

[9] R. Su, S. A. Ermiliov, A. V. Liopo, A. A. Oraevsky, presented at Photons Plus Ultrasound: Imaging and Sensing 2012 **2012**.

[10] J.-H. Lee, Y.-M. Huh, Y.-w. Jun, J.-w. Seo, J.-t. Jang, H.-T. Song, S. Kim, E.-J. Cho, H.-G. Yoon, J.-S. Suh, *Nature medicine* **2007**, 13, 95.
